# Consumer Co-Design of an Online Resource to Build Communication Skills of Health Consumers: Mixed Methods Study

**DOI:** 10.2196/77263

**Published:** 2025-12-12

**Authors:** Alison Beauchamp, Julieanne Hilbers, Natali Cvetanovska, Anna Wong Shee, Lidia Horvat, Sandra Rogers, Andrea Cooper, Elizabeth Flemming-Judge, Sue Rawlinson, Rebecca Jessup

**Affiliations:** 1 School of Rural Health, Monash University, D501, Level 535 Rainforest Walk, Wellington Road, Clayton, Victoria, 380035, Australia, +61 399051327; 2 Compassionate Communities, Perron Institute, University of Western Australia, Nedlands, Western Australia, Australia; 3 Office of Research, Northern Health, Melbourne, Australia; 4 School of Medicine, Deakin University, Melbourne, Australia; 5 Research Development, Grampians Health, Ballarat, Australia; 6 Department of Health, Safer Care Victoria, Melbourne, Australia; 7 Patient Experience Office, Monash Health, Melbourne, Australia; 8 Department of Health, Health Consumer, Safer Care Victoria, Melbourne, Australia; 9 Health Consumer, Community Partnerships, South East Sydney Local Health District, Sydney, Australia

**Keywords:** patient-provider communication, patient understanding, health literacy, co-design, consumer engagement, user-centered design, stakeholder collaboration

## Abstract

**Background:**

Information provided by health professionals can be complex and is often not well understood by health care consumers, leading to adverse outcomes. Clinician-led communication approaches such as “teach-back” can improve consumer understanding, yet are infrequently used by clinicians. A possible solution is to build consumers’ skills to proactively check their understanding rather than waiting for the clinician to do so; however, there are few educational resources to support consumers in building these skills.

**Objective:**

This study aimed to co-design a web-based learning resource for consumers to check they have understood information provided by a clinician (ie, to “check-back”).

**Methods:**

This mixed methods study used a co-design approach, consisting of 2 phases. The study was conducted during the COVID-19 pandemic, and all activities were conducted online, via email or telephone. Phase 1 (needs assessment) involved first establishing an Expert Panel of consumers, clinicians, and academic experts to guide all co-design steps of the study. Next, we sought to understand issues around health communication through focus groups and interviews with consumers and clinicians. Participants were recruited from outpatient settings and consumer representative programs within 3 health services in Victoria, Australia. Focus groups and interviews aimed to identify factors that might influence consumers’ use of check-back. Deductive analysis based on the Capability, Opportunity, and Motivation-Behavior (COM-B) model was used to identify initial themes; these were discussed in depth with the Expert Panel and barriers within each theme identified. A rapid literature review was undertaken to identify strategies for web-based communication training for consumers. Phase 2 (creation of the online resource) involved an iterative process. In an online meeting, Expert Panel members brainstormed ideas for addressing barriers and prioritized these ideas for inclusion in the resource. Several drafts of the content were written before a draft online version was built. This draft was reviewed by the Expert Panel, who recommended extensive revisions. Following these revisions, we conducted an online survey and focus group with consumers and clinicians from Phase 1 to identify further improvements. Findings from this consultation were used to make final changes to the online resource.

**Results:**

The Expert Panel included 12 members. Phase 1 focus groups and interviews were held with 39 consumers and 16 clinicians. Five themes were identified: self-efficacy, pre-existing skills, clinician attitudes, information complexity, and internal barriers such as embarrassment. Phase 2 survey and focus group participants identified several issues with the second draft of the resource, focusing on functionality, accessibility, and layout. Usability and acceptability of the resource were rated highly by participants.

**Conclusions:**

Findings highlight the value of using co-design to develop a consumer-centered, web-based learning resource. Further evaluation is required to demonstrate its effectiveness at improving consumer understanding.

## Introduction

The health system places a high burden on health care consumers to understand and apply health information across a range of contexts. From providing informed consent to following complex health care regimes or participating in decisions about their health, it is important that people are able to understand and recall information given to them by health professionals [1]. Studies have demonstrated that health information is frequently not understood or remembered by health care consumers [2-5], leading to increased treatment burden and adverse health outcomes [6-8]. Factors that may affect understanding originate from both the consumer and health professional and include limited health literacy and confidence in asking questions or engaging in health care discussions [3,5,9-11], and limited ability of the health professional to convey health information clearly [7,11-13]. Evidence-based approaches to address this communication gap are required.

One communication approach that has been associated with improved consumer understanding and recall across diverse settings and health conditions is “teach-back” [14-17]. This approach involves a health professional providing information and then confirming the consumer’s understanding by asking them to explain back that information in their own words. In this way, any misunderstandings can be identified and addressed within an active, 2-way interaction. There is strong evidence that checking health care consumers’ understanding through approaches such as teach-back can build greater rapport and more active communication [18], and may lead to improved knowledge, skills, and self-care abilities [14-16]. Yet despite the fact that these approaches offer a simple and effective approach for improving understanding, they are not widely adopted by health professionals [19]. Approaches such as teach-back rely on the health professional to initiate the process, and there are several barriers to their use, including lack of confidence and concern about the consumer reaction [19]. Despite this, evidence suggests that health care consumers are receptive to the idea of being asked to confirm their understanding, stating this would help with their learning and support them to remember important information [18,20].

One possible strategy to increase the use of approaches such as teach-back is to build consumers’ skills and confidence to actively check their understanding rather than waiting for the health professional to do so. For example, this may involve the consumer saying, “Can I just check that I’ve understood everything. Did you say....” Encouraging consumers to initiate the process of repeating back information using their own words (ie, to “check-back”) may be a feasible approach to improve understanding and recall of information across many health care settings. However, while some communication skills, such as asking questions and giving information, may come naturally to many health care consumers, initiating the process of checking-back requires more advanced skills [21]. These include the ability to actively listen, to summarize key information, and then the confidence to explain back that information in their own words [21].

Many of these skills could be learned through communication training or coaching; these learning approaches have been demonstrated to be effective in increasing consumer participation and engagement in health care interactions [21-23]. However, while education-based interventions may provide health care consumers with the skills to initiate check-backs themselves, there are also many barriers to consider, including low self-efficacy and confidence, perceived power imbalance, the communication style of the health professional, cultural factors, and structural factors such as short consultation times [24]. Any intervention that supports health care consumers to use the check-back process needs to address these barriers while also considering the context within which the intervention will be used. An effective methodology for developing interventions that are responsive to local barriers is that of co-design. The co-design approach in health enables equal involvement of consumers, health professionals, and other stakeholders throughout all stages of intervention development, ensuring that the end product meets the needs of those for whom it is designed [25-27]. Co-design can be defined as an active collaboration between stakeholders in designing solutions to a prespecified problem and is shown to be an effective approach for developing strategies to improve health communication [28-31].

This paper describes the co-design of a web-based learning resource for health care consumers. The decision to select a web-based approach was initially driven by consumers who had been involved in co-design of a similar web-based learning resource for clinicians called “teach-back” [32]. Those consumers noted they would like to see a web-based learning resource specifically for consumers that prompted them to initiate the teach-back process (ie, to “check-back”). The aim of this web-based learning resource is therefore to equip consumers with the necessary skills and confidence to initiate the check-back process in any setting and interaction with a health professional, that is, to confirm understanding through summarizing and repeating back key information to the health professional. For the purposes of this paper, the term “consumers” will hereafter be used to refer to health care consumers, patients, or users of the health system.

## Methods

### Study Design

**Figure 1.**
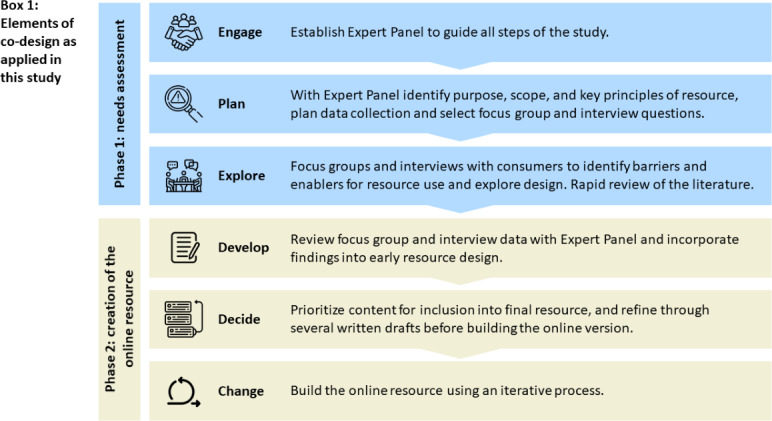
Overview of the study phases and co-design elements in the study. Icons made by Uniconlabs, rukanicon, photo3idea_studio, Hilmy Abiyyu A., juicy_fish, and Saepul Nahwan from Flaticon [36].

This mixed methods study uses a co-design approach developed by Boyd et al [33,34]. This approach has six elements: (1) engage, (2) plan, (3) explore, (4) develop, (5) decide, (6) change. As noted above, co-design seeks to develop solutions to an identified issue through first understanding consumer perspectives about that issue. Accordingly, the first 3 elements of this framework involve collaborating with key stakeholders to establish the study parameters and explore consumer experiences in depth; we have called this the “needs assessment” phase. The second 3 elements aim to improve that experience through deciding what to improve and then taking action that leads to change; we have called this phase “creation of the online resource.” Within this framework, elements can overlap and their order may vary. Our application of this framework is shown in Figure 1. We adhered to the Guidance for Reporting Involvement of Patients and the Public (GRIPP2) to report consumer involvement in this study [35].

### Phase 1: Needs Assessment

#### Engage and Plan

The needs assessment phase followed the first 3 elements of the Boyd co-design approach (engage, plan, and explore). “Engage” refers to establishing and maintaining meaningful relationships with consumers and clinicians [33]. This was achieved by establishing a co-design team, which we named the “Expert Panel” in recognition of the lived experience and expertise of Panel members. Purposive sampling was used to select Expert Panel members from key stakeholder groups, including health care consumers, clinicians, and academic experts. Consumer members were invited through an expression of interest circulated within the Consumer Branch of the Victorian government agency for quality and safety in health care (Safer Care Victoria) and were remunerated for their involvement. Inclusion criteria for consumers were having lived experience as a health care consumer and being aged 18 years or older. Clinicians and academic experts with experience in health literacy, health communication, and co-design were invited through previous collaborations and networks.

The purpose of the Expert Panel was to guide all co-design aspects of the study. The Expert Panel initially met in October 2020 to agree upon terms of reference for the group, including roles, responsibilities, and frequency of meetings. In this online meeting, planning for the project was also undertaken, including clarifying the overall purpose and scope of the learning resource and developing a plan for the “explore” step of the study. It was determined by the Expert Panel that “explore” should include 2 aspects: interviews or focus groups with consumers and clinicians to identify potential barriers to using check-back within health care interactions, and a rapid literature review of studies addressing communication skills training for consumers.

#### Explore

##### Setting and Participants

The “explore” element of this study was conducted across 3 health services in Victoria, Australia with 1 service in a regional setting and 2 in Metropolitan Melbourne. Within the regional service, participants were recruited from the community health service, specifically outpatient rehabilitation programs and chronic disease outpatient clinics. Within the Metropolitan services, participants were recruited from chronic disease outpatient clinics and from the hospital volunteer or consumer representative programs. Of note, the 2 Metropolitan health services support communities in the outer north and south-east of Melbourne, with high proportions of culturally and linguistically diverse populations.

Convenience sampling was used to recruit consumers and clinicians from participating health services to take part in focus groups and interviews. For consumers who were clients of outpatient rehabilitation programs and chronic disease outpatient clinics, client lists were screened and those eligible were contacted by phone or email to discuss the study. For consumer representatives and hospital volunteers, email alerts were sent and those who were interested were invited to contact the researchers. Clinicians included nursing and allied health professionals from the above sites, who were invited to a focus group or interview via email. Consumers and clinicians who participated in focus groups or interviews were also invited to take part in consultation activities in Phase 2. Inclusion criteria for consumers and clinicians were being aged 18 years or older, with the capacity to provide informed consent.

##### Focus Groups and Interviews With Consumers and Clinicians

Data were collected via focus groups or semistructured interviews for participants who were unable to take part in a focus group. Focus groups were held separately for consumers and clinicians. Due to COVID-19 restrictions, all focus groups were conducted via teleconference and interviews via telephone. Focus groups and interviews were divided into 2 parts, with the first part aiming to identify factors that might influence consumers’ use of the check-back process with health professionals. To provide context for participants, short “vignettes” were written describing challenges that a consumer may face when using check-back. These vignettes were based on a synthesis of patient experience stories from one of the participating health services and were used to “depersonalize” discussions and ensure participants felt comfortable in sharing their ideas without needing to refer to their personal experiences (refer to Multimedia Appendix 1 for an example of a vignette and focus group and interview guide).

**Figure 2.**
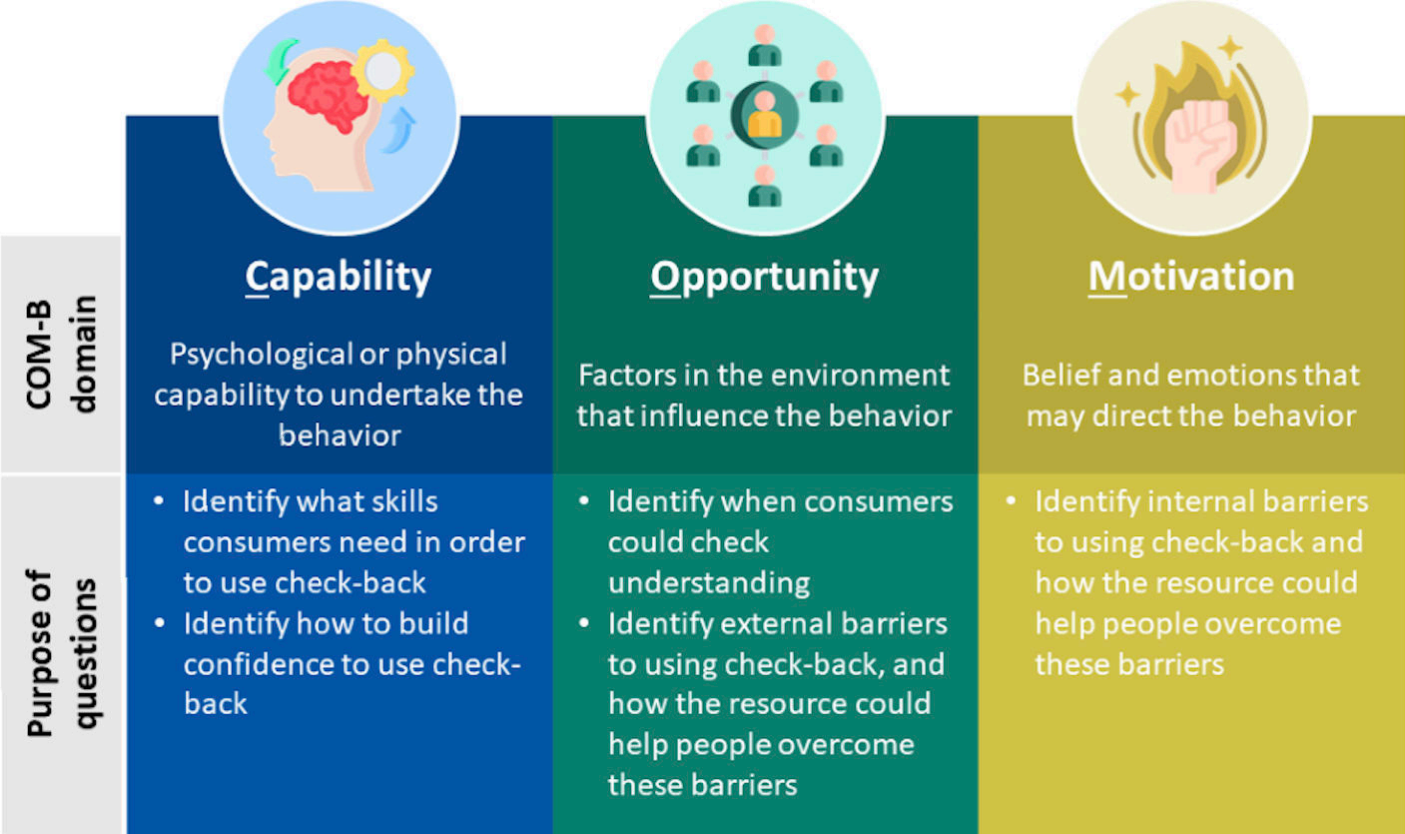
Domains of the COM-B model and their application to focus group and interview topics to identify potential barriers to using check-back within health care interactions. COM-B: Capability, Opportunity, and Motivation-Behavior. Icons made by Uniconlabs, Freepik, and pojok d from Flaticon [36].

A series of seeding questions to prompt discussions was developed using the Capability, Opportunity, and Motivation-Behavior (COM-B) model which provides a theoretically-based approach for developing behavior change interventions [35, 37]. The COM-B model can be used to identify skills and abilities that an individual needs in order to adopt a behavior, as well as the environmental conditions that might support adoption of that behavior. For this study, the behavior was consumer-initiated check-back—that is, confirming understanding through summarizing and repeating back key information to the health professional. The COM-B model considers 3 key domains associated with behavior change: Capability, Opportunity, and Motivation [38]. Figure 2 shows examples of topics used to explore participants’ views, based on these 3 domains.

Focus group and interview participants were also asked for ideas about the design of the web-based resource, including how it could be made accessible for consumers. These included the types of scenarios to show in the videos and strategies to ensure the resource was easy to use.

All vignettes and topics for the focus groups and interviews were discussed with the Expert Panel to ensure they were appropriate and would be easily understood by participants. Focus groups and interviews were audio-recorded and transcribed. Deductive thematic analysis was used to analyze the data and identify patterns (themes) within the data that could be used to develop content for the resource [39]. Data were analyzed by 2 researchers (AB and JH). Transcripts were read several times to become familiar with the data and to generate an initial set of themes using the 3 domains of the COM-B model as the parent themes (Capability, Opportunity, and Motivation). The themes that arose from this initial analysis were discussed in depth during an online meeting with the Expert Panel in order to gather their insights into the data, thus providing a more balanced approach to analysis. Barriers and enablers within each theme were subsequently identified by JH and AB, and then cross-checked with the Expert Panel via email. Quotes that accurately illustrated the themes were also identified.

##### Literature Review

The needs assessment was also informed by a rapid review of the literature (Multimedia Appendix 2). Briefly, this review aimed to identify best-practice strategies for web-based communication skills training directed at consumers. A narrative approach was used to synthesize the data. Recommendations for best-practice strategies from this review were provided to the Expert Panel for consideration during development of the web-based resource.

### Phase 2: Creation of the Online Resource

#### Develop and Decide

The second phase involved creating the web-based learning resource, following the 3 elements from Boyd co-design framework of develop, decide, and change. For the development and decision steps, findings from the interviews and focus groups and the rapid literature review were discussed with the Expert Panel. In this meeting, identified barriers and enablers to using the check-back process were considered, and the Expert Panel was asked to generate ideas for how these could be addressed in the web-based resource. Ideas were generated by brainstorming through facilitated discussion, a recognized approach in co-design [40-42]. Each theme, barrier, and enabler identified from the focus group and interview data was discussed in turn, with the goal being to identify how each could be targeted within the resource; for example, whether a video-based scenario could be used to demonstrate how to address a specific barrier. The proposed content was then prioritized for inclusion by the Expert Panel. There were no criteria for inclusion of this content, and decisions were made through discussion. Based on these priorities, several written drafts of the resource content were developed and refined, each in collaboration with the Expert Panel.

#### Change

##### First Draft of the Resource

The change step involved building an online version of the learning resource using an iterative process. As a first step, audio and video content were recorded over 2 days at one of the participating health services. Four scenarios were filmed with volunteer patients and health professionals acting out a scripted health care interaction. An additional 16 short video clips and brief audio recordings were also recorded, all of which were based on example scenarios and quotes provided by Phase 1 focus group and interview participants. A complete first draft of the web-based resource was then built during April-May 2021. This draft version was reviewed by the Expert Panel who recommended extensive revisions related to functionality, accessibility, and layout. These recommendations are shown in Table 1, with the corresponding revisions that were made.

**Table 1.** Summary of identified issues for drafts 1 and 2 of the check-back resource, and examples of revisions made.

Draft and issue category	Identified issues	Example of revisions made
Draft 1 (reviewed by Expert Panel)		
Functionality and navigation	Resource is difficult to navigate Not able to move freely through the resource Not clear how to access videos	Include an overview and a progress bar Allow progression without needing to view videos Provide clearer instructions for using videos and audio
Accessibility	Difficult for hearing or sight-impaired consumers to use Limited representation of diversity	Add captions to videos and include audio buttons Increase font size and color contrast Imagery changed to show more diversity
Layout and length	Not clear what check-back is at the start Not all videos are relevant to everyone, which makes the resource overcomplicated Overall resource too long and hard to follow	Show steps in check-back earlier Have 1 main video demonstrating check-back and the others as optional viewing Group content with the most important elements first
Draft 2 (reviewed by consumers and clinicians)		
Functionality and navigation	Clearer instructions for navigating to the next page are needed Wording of some instructions about using the resource is ambiguous	Size of “Next” button increased and instructions simplified Clarify instructions and use consistent language throughout
Accessibility	Links to written transcripts needed Audio buttons not easily visible More pictures of families needed	Provide links to a “resources” page with transcripts Increase size of the audio button Imagery changed to include families
Layout and length	Home page text is not grouped next to relevant imagery and is easily missed	Move some text on the home page to be more obvious and use bold text for emphasis

##### Second Draft of the Resource

Following these revisions, we consulted with consumers and clinicians to determine the usability, acceptability, and perceived value of draft 2 of the resource and to identify areas for further improvement. Participants were those who had taken part in Phase 1 focus groups and interviews and were willing to take part in this stage. Two approaches were used for this consultation: (1) an anonymous online survey of consumers and clinicians and (2) a focus group with consumers from 1 participating health service. For the online survey, respondents were asked to rate usability (ease of use and navigation), acceptability (whether the resource length was appropriate and overall quality of the module), and perceived value (how interesting the content was and whether the videos clearly showed how to use check-back). A 5-point Likert scale (from strongly disagree to strongly agree) was used. Participants were also asked to rate whether they would recommend it to others (5-point Likert scale; definitely yes to definitely no). An open-text response option was provided for additional comments, including recommendations for any revisions. Data from the Likert scale responses were reported as proportions, and open-text responses were collated. The 60-minute focus group was held with consumers via teleconference, with a link to the learning resource sent to participants ahead of time. During the focus group, researchers presented each section of the resource and asked participants about usability, acceptability, and perception of value, using the questions asked in the online survey to frame the discussion. Participants were also invited to make recommendations for any revisions. The focus group was audio-recorded and transcribed. Data from the survey and focus group were used to make final changes to the online resource in consultation with the Expert Panel (Table 1).

### Ethical Considerations

Ethics approval for the use of patient stories and to conduct all focus groups and interviews and filming was provided by the Research Ethics Committees of Monash Health, Northern Health, and Grampians Health Service (Ethics Review Manager reference numbers: 68367, 68046, and 62381). All participants provided written informed consent for focus groups and interviews and implied consent for the online survey. All study data were deidentified for analysis and reporting purposes, and no identifying data are reported. Expert Panel members were compensated for their time based on Safer Care Victoria’s Remuneration for Partners Policy. Consumers who attended focus groups and interviews were offered a AUD $20 (US $14) gift voucher.

## Results

### Results From Phase 1: Needs Assessment

#### Expert Panel

The Expert Panel included 4 consumers with extensive lived experience of health care. There were 4 clinician members (including 1 medical specialist and 3 allied health or nursing professionals), and 4 experts in health communication, health literacy, or co-design. One general practitioner (GP) was invited to participate but was only able to provide input into 1 round of email consultation. The Expert Panel represented a diverse range of perspectives across age, gender, and culture and guided all stages of the study through 4 online meetings and 2 rounds of email consultations.

#### Focus Groups and Interviews

Focus groups or interviews were held with 39 consumers and 16 clinicians; specifically, 3 consumer (n=14) and 2 clinician (n=11) focus groups, and 25 consumer and 5 clinician interviews. Among 39 consumers, ages ranged from late 20s to early 80s, and there were 12 males (31%). Out of 39, 10 consumers (26%) were born outside Australia or spoke a language other than English at home, with 2 of these consumers interviewed in Arabic by an accredited qualified interpreter. Among clinicians, roles included nursing, pharmacy, occupational therapy, and physiotherapy. Table 2 presents findings from Phase 1 focus groups and interviews. Under the 3 COM-B domains, 5 themes were identified: self-efficacy and confidence, pre-existing skills, health professional behaviors and attitudes, information complexity and overload, and internal barriers such as embarrassment. For each theme, barriers and enablers are shown, each illustrated by a quote.

**Table 2.** Themes, barriers, and enablers to using check-back within health care interactions as identified from focus group and interview data with consumers (n=39) and clinicians (n=16).

Domain and theme (frequency)	Barriers and enablers from focus groups and interviews	Example quotes
COM-B domain: Capability		
Self-efficacy and confidence (7 consumers, 2 clinicians)	Many people would find it difficult to ask clinicians to repeat information. The resource could include messages that aim to counteract shyness or beliefs about hierarchy and highlight how common it is for people to not understand information.	“Have you heard it before that older people have them on a pedestal and are a bit timid on asking questions?” (Client #4) “People need to be encouraged to think that you can ask questions you can double check...because it’s quite normal to not understand.” (Volunteer #4)
Pre-existing skills (9 consumers, 1 clinician)	People may already have the skills to check understanding as they often do this in everyday life.	“Yes, I have a bad memory for names. When someone gives me their name, I’ll repeat it back to them.” (Volunteer #2)
COM-B domain: Opportunity		
Health professional behaviors and attitudes (9 consumers, 2 clinicians)	Clinicians’ behavior can limit a consumers’ confidence and empowerment, especially if they appear authoritative or disengaged. The resource could emphasize the consumers’ right to ask questions and to clarify information.	*“*...and they have got the attitude of them and us, it’s like well what do you know, I’m the specialist.” (Client #1) “We have to empower consumers to have the confidence to do that in a non-confrontational way and I think that perhaps the videos would assist with that.” (Consumer representative #1)
Information complexity and overload (11 consumers)	Clinicians frequently use complex terms, and the amount of information can be overwhelming and difficult to remember. Include tips such as asking the doctor to write things down. Highlight the value of bringing a support person with you.	“That’s often the problem, too many abbreviations and jargon is used.” (Consumer representative #2) “I ask my doctor if he minds jotting down those points that he highlighted so I can have something once I’m out the door, something to ponder on.” (Volunteer #6)
COM-B domain: Motivation		
Internal barriers (20 consumers, 2 clinicians)	Internal factors include embarrassment or lack of confidence. Consumers may be overwhelmed by the situation and by emotions such as worry or fear. The resource could emphasize the benefits of understanding information and include messages that give “permission” to clarify understanding.	“You’ve got patients waiting outside, I don’t want to hold the doctor up by asking silly questions because I don’t understand.” (Volunteer #7) “That no question is a silly question. …we’d rather you understand and question, than go home and do the wrong thing.” (Client #4)

#### Literature Review

From the rapid literature review (Multimedia Appendix 2), 15 relevant recommendations were identified. These were presented to the Expert Panel for consideration during development of the check-back resource. Key recommendations included the importance of raising consumers’ awareness about their role in health communication and using approaches to build self-efficacy in communication, for example, demonstrate behaviors through video clips of peers. Text font should be large and clear with consistent layouts throughout, and text should be brief and jargon-free. For culturally diverse populations, the use of visual aids and including people from various cultural groups is recommended, as is the use of transcripts of any video commentary to support people with a hearing impairment. A mix of modalities is effective for the delivery of information; these include infographics, videos, text, animation, and narration. Including an interactive component can increase user engagement, and the addition of additional printed resources, such as question prompt lists, is also recommended.

### Results From Phase 2: Creation of the Web-Based Resource

The check-back resource was built through an iterative process. As described in the “Methods”, the Expert Panel recommended extensive revisions to the first complete draft of the resource (Table 1); a second complete draft was then developed, followed by a further round of consultation with consumers and clinicians through an online survey and focus group.

#### Online Survey

Out of 55, 11 (20%) respondents completed the online survey. Table 3 shows a summary of survey findings relating to usability, acceptability, and perception of value.

**Table 3.** Findings from the online survey to determine usability, acceptability, and perceived value of the check-back resource (n=11 clinicians and consumers).

Question	Response 1, n (%)	Response 2, n (%)	Response 3, n (%)	Response 4, n (%)	Response 5, n (%)
The resource was...^a^					
Easy to use	0 (0)	2 (18)	0 (0)	3 (27)	6 (55)
Easy to navigate	0 (0)	2 (18)	1 (9)	1 (9)	7 (64)
Interesting	0 (0)	1 (9)	0 (0)	5 (45)	5 (45)
Right length	0 (0)	1 (9)	3 (27)	3 (27)	4 (36)
Videos showed check-back (n=10)	0 (0)	0 (0)	0 (0)	4 (40)	6 (60)
How would you rate the quality of the module^b^?	0 (0)	1 (9)	0 (0)	4 (36)	6 (55)
Would you recommend this module to others for learning about check-back^c^?	0 (0)	1 (9)	0 (0)	4 (36)	6 (55)

a Responses: 1=Strongly disagree, 2=Somewhat disagree, 3=Neither, 4=Somewhat agree, and 5=Strongly agree.

b Responses: 1=Extremely bad, 2=Somewhat bad, 3=Neither good nor bad, 4=Somewhat good, and 5=Extremely good.

c Responses: 1=Definitely not, 2=Probably not, 3=Might or might not, 4=Probably yes, and 5=Definitely yes.

Overall, most participants agreed or strongly agreed that the resource was easy to use (9/11, 82%) and navigate (8/11, 71%), the content was interesting (10/11, 90%), and the videos demonstrated how to use check-back (10/10, 100%). Most participants (10/11, 91%) rated the quality of the module as somewhat or extremely good and said they would recommend it to others (10/11, 91%). One respondent said they would not recommend it, commenting that there were too few videos demonstrating what check-back is. Open-text comments revealed that the resource was very engaging, with suggestions for improvement mostly focused on enhancing the navigability of the resource.

#### Consumer Focus Group

**Figure 3.**
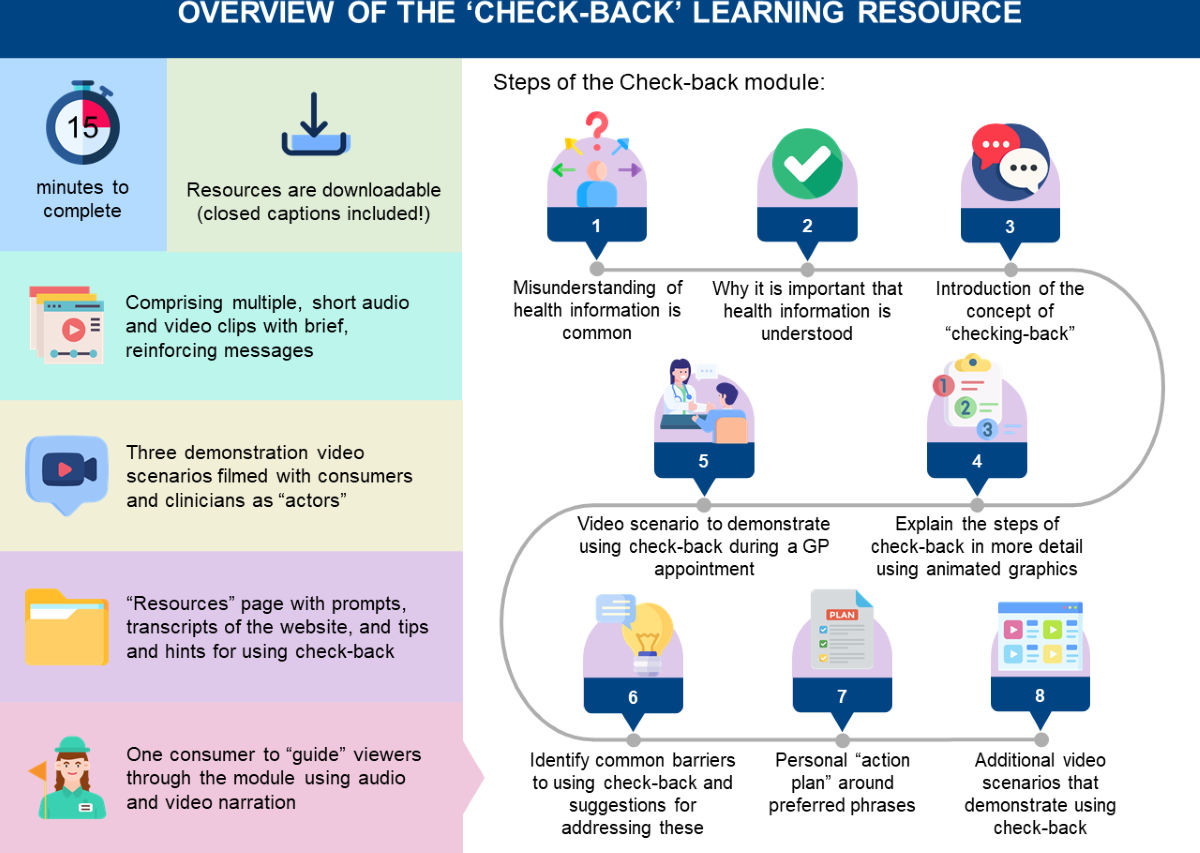
Overview of the “check-back” learning resource [43]. GP: general practitioner. Icons made by Saepul Nahwan, Becris, Freepik, PixelVerse, Roundicons, smashingstocks, and vectorsmarket15 from Flaticon [36].

Comments on draft 2 received from the consumer focus group (n=5) also focused on navigation, including the need for clearer links to audio and written transcripts. Minor revisions to images and text were also recommended, including more use of family images. In response to these findings and those from the online survey, additional revisions to the resource were made in collaboration with the Expert Panel (Table 1). The final resource was approved by the Expert Panel in October 2021 and made publicly available from April 2022. An overview of the check-back resource is shown in Figure 3.

## Discussion

### Principal Findings

This study describes the development of a web-based learning resource to support consumer-driven check-back, using a co-design approach to partner with key stakeholders, including consumers with lived experience and health professionals. Early feedback suggests that the resource is engaging and accessible for consumers, with content that is interesting and relevant. In part, this relevance may be due to the use of the co-design approach with its focus on embedding the perspectives of consumers and other end users throughout the design and development process. Co-design has become increasingly used in health care to undertake service evaluation and quality improvement activities [25]. However, few studies have described using co-design in patient-provider communication, especially those that engage consumers as a core component of the process or that develop web-based resources [28,30,44]. One study used co-design to address communication barriers for patients with heart failure, resulting in a paper-based question prompt list for patients [28]. In another study, a communication training package targeted toward radiographers was co-designed with patients with breast cancer [30]. This study adds to the limited evidence for the value of using co-design to develop consumer-oriented web-based resources, including those aiming to assist consumers to build their communication skills in any healthcare setting or with any clinician.

Challenges that have been identified with using co-design include the exclusion of people with lived experience from the process, having the methods established before beginning the project, and not allowing for equal partnership between consumers, health professionals, and researchers in the design process [45]. In this study, we aimed to ensure that consumers and health professionals guided the content and design of the resource, thus providing an essential “end user” perspective. Through active and considerate listening and following an iterative process that remained focused on a clear end product, we adhered to the 5 core principles for successful co-design: being inclusive, respectful, participative, iterative, and outcomes-focused [46].

This study also adds to the limited evidence describing web-based communication training resources for consumers. A 2017 systematic review of the literature on training consumers in communication skills identified that just 5 of the 32 included studies used web-based training [21]. These included 3 randomized controlled trials: a web-based intervention to increase consumers’ communication self-efficacy [44], an interactive website that used consumer narratives to prepare participants for communicating with doctors [47], and a consumer and physician training intervention comprising multiple demonstration videos [48]. Overall, these studies demonstrated that web-based interventions that assist consumers to improve communication are effective at increasing their satisfaction and skills [44,47,48], and at supporting them to achieve treatment-related goals [23]. While the check-back resource has not yet been formally evaluated, these studies identify aspects of evaluation that may be relevant, including consumer satisfaction with the resource, whether it results in behavior change within health care interactions (such as increased confidence to ask questions), and whether it is effective at increasing understanding and recall in the short and long term.

The final check-back learning resource is multimodal in its delivery. Many of its features have been shown in other studies to be effective at improving consumer understanding, including the use of videos to model behavior [49], infographics [50], animations [51], and guides or narrators [52]. The use of multiple modes of delivery in the check-back resource may also address the diversity of learning styles found within any population [53]. This may be particularly relevant for consumers with lower health literacy who are often more likely to experience poor health care communication [12,53]. Web-based learning using a mix of modalities (including images) has been shown to be feasible and effective among people with low health literacy [54], and may also be useful for people with cognitive challenges, or those who do not speak the local language. Digital health literacy (defined as the ability to seek, find, understand, and appraise health information from electronic sources, and to apply that knowledge to solve health-related problems) [55] can also be enhanced by resources that are easily accessible and usable [56].

In terms of future steps, tailoring of the check-back resource to particular cultural groups will be required, as evidence shows that ethnic and racial minorities receive suboptimal health communication [21], are less likely to ask questions [57], and may frequently misunderstand information [58]. However, cultural tailoring of this resource will involve more than linguistic translation as cultural values, norms, or beliefs need to be fully considered [58,59]. Use of the co-design approach to ensure cultural relevance in future adaptations is warranted. Other adaptations may include creating versions of check-back that are accessible for consumers with few digital skills or limited access to the internet. Given the known digital divide in high-income countries, it is important to provide alternative approaches for consumers to learn about check-back to enhance equity and accessibility [60.

Finally, it is important to acknowledge that addressing the problem of poor health communication by focusing only on building consumer skills will be insufficient. Health and community providers and policy makers may consider introducing this resource to consumers as part of a suite of multilevel strategies aimed at building organizational responsiveness to the communication and health literacy needs of their consumers [61,62].

### Limitations

To our knowledge, this is the first study to co-design a web-based, interactive education resource for consumers to improve their ability to communicate effectively during health care interactions. There were some limitations with this study, including the limited representativeness of the sample for the needs assessment phase, as participants were from Victoria, Australia only. Recruitment was also limited by COVID-19 restrictions, and therefore, we needed to include a number of consumer representatives who may have been more empowered than a “typical” health care consumer. In addition, information on the number of consumers and clinicians that were approached to participate in focus groups was not consistently available, and therefore, we cannot provide participation rates. Participants were primarily English-speaking only, although several culturally and linguistically diverse consumers were included in focus groups and interviews. While the Expert Panel included a medical specialist, there was only limited input from a GP. Given that consumers are most likely to use check-back with their GP, this may have affected the identification of barriers to its use.

### Conclusions

The issue of poor communication in health care is well-recognized, and innovative strategies are required, including those that enable consumers to participate in active 2-way communication. Through the use of co-design with key stakeholders, we developed an accessible, consumer-centered learning resource that may have high applicability and utility for consumers across a broad range of health and community settings. This study provides a starting point for future research into consumer-led initiatives to improve communication, including for population groups known to have significant challenges in this area.

## Supplementary material

10.2196/77263 Multimedia Appendix 1 Example of a vignette used in focus groups and interviews, and focus group and interview guide.

10.2196/77263 Multimedia Appendix 2 Consumer check-back: a rapid literature review to inform development of an online learning resource for consumers.

## Abbreviations

COM-B: Capability, Opportunity, Motivation-Behavior
GP: general practitioner
GRIPP2: Guidance for Reporting Involvement of Patients and the Public
