# Supplementary material for: Consumer Co-Design of an Online Resource to Build Communication Skills of Health Consumers: Mixed Methods Study
Source: JMIR Form Res. 2025 Dec 12;9:e77263. doi: 10.2196/77263 (PMC12700338; doi:10.2196/77263)
Supplement: Multimedia Appendix 1 [file formative-v9-e77263-s001.docx]

## **Supplementary material 1**

### Example of a vignette used in focus groups/interviews, and focus group/interview guide

### I’m just going to read a short story about a situation where someone didn’t understand something clearly. I’ll read it through, then ask you a few questions about it.

**Vignette**

Maria is in her 60’s and migrated to Australia 15 years ago. She recently saw her GP about some shortness of breath she’d been having. The GP thought that Maria might have a heart problem, and told her that she’d need 3 or 4 different tests, and would also need to start on some new medication. Maria was quite concerned to hear something might be wrong with her heart, but when the doctor asked if she had any questions, she couldn’t think of anything to ask. She felt was there was a lot of new information to try and take in during the visit, and left the appointment not really clear about she was supposed to do next.

**Questions**

1. What are some of the reasons that Maria might not have completely understood what was going on?

What sort of tips could you give to people like Maria to help them better **understand** what a doctor tells them? What do you use?

What sort of advice would you give to people like Maria to help them **remember** what a doctor tells them?

*I’m now going talk about something called Teach-back. Teach-back means explaining back information that someone has just given you, so you can be sure that you’ve understood it clearly. We can do this when we’re in a health care setting, such as at the GPs or in hospital. This could help us to really understand clearly what we needed to do. Coming back to Maria for an example of this:*

After her last visit to the GP, Maria made a promise to herself that she would no longer walk away from health appointments not really knowing what is going on. She now uses teach-back in every appointment to double-check that she has understood what she’s been told. She’s developed a trick she uses where she says to the Dr “Let me make sure I understand – Each morning, I should……” (whatever the doctor has told her).

**Questions**

1. How useful would it be for patients to say this in health appointments?
2. Is using teach-back to check that you’ve understood something you could imagine yourself doing? That is, to say to a health professional “Let me make sure I understand…” or “I just want to check I’ve got everything you told me. Did you say….”
   1. If so, how would you phrase it, what would you say to the health professional?
   2. How easy or difficult would it be for you to say this?

### We want to develop a website that teaches patients to feel more confident when talking with health professionals, and to encourage patients to clarify they’ve got something by using teach-back so that they walk away from health appointments understanding what is going on. I just want to ask you some questions about this.

**Questions**

1. Is a website like this something you would use – why or why not?
2. What should we be careful NOT to say or do?
3. What would you like to see on a website like this?
