# Supplementary material for: Consumer Co-Design of an Online Resource to Build Communication Skills of Health Consumers: Mixed Methods Study
Source: JMIR Form Res. 2025 Dec 12;9:e77263. doi: 10.2196/77263 (PMC12700338; doi:10.2196/77263)
Supplement: Multimedia Appendix 2 [file formative-v9-e77263-s002.docx]

**Supplementary File 2**

**Consumer Check-back: A rapid literature review to inform development of an online learning resource for consumers**

**Table of Contents**

[BACKGROUND 6](#_Toc54550319)

[AIM 7](#_Toc54550320)

[METHODS 7](#_Toc54550321)

[Search strategy 7](#_Toc54550322)

[Inclusion criteria 8](#_Toc54550323)

[Exclusion criteria 8](#_Toc54550324)

[Data extraction and analysis 8](#_Toc54550325)

[RESULTS 8](#_Toc54550326)

[1. Patient-Provider Communication 9](#_Toc54550327)

[1.1 Summary of evidence: 9](#_Toc54550328)

[1.2 Influences and barriers to effective patient-provider communication 9](#_Toc54550329)

[1.3. Importance of including consumer/patient perspectives on communication 10](#_Toc54550330)

[2. Skills Training For Patient-Provider Communication 10](#_Toc54550331)

[2.1 Summary of evidence: 10](#_Toc54550332)

[2.2 Interventions delivered to patients only 10](#_Toc54550333)

[2.3 Interventions delivered to both patients and clinicians 11](#_Toc54550334)

[3. Computer Aided Learning in Healthcare 11](#_Toc54550335)

[3.1 Summary of evidence: 11](#_Toc54550336)

[3.2 What is Computer Aided Learning (CAL)? 11](#_Toc54550337)

[3.3 Is CAL effective for patient learning? 11](#_Toc54550338)

[3.4 Is CAL effective for communication training? 12](#_Toc54550339)

[4. Recommended Design & Delivery Elements of CAL 12](#_Toc54550340)

[4.1 Summary of evidence: 12](#_Toc54550341)

[4.2 Delivery modes and layout 12](#_Toc54550342)

[4.3 Importance of interactivity 13](#_Toc54550343)

[4.4 Additional considerations in design 13](#_Toc54550344)

[5. Value of Adding Printed Resources 14](#_Toc54550345)

[5.1 Summary of evidence: 14](#_Toc54550346)

[5.2 Question Prompt lists – should these be included as an additional resource? 14](#_Toc54550347)

[5.3 Fact sheets/Frequently Asked Question (FAQ) sheets 15](#_Toc54550348)

[6. Visual Communication Tools 15](#_Toc54550349)

[6.1 Summary of evidence: 15](#_Toc54550350)

[6.2 Value of videos to model behaviours and provide information 15](#_Toc54550351)

[6.3 Infographics or pictograms 15](#_Toc54550352)

[6.4 Graphs & numbers 16](#_Toc54550353)

[7. Use of stories or narratives to deliver messages 16](#_Toc54550354)

[7.1 Summary of evidence: 16](#_Toc54550355)

[7.2. Stories and narratives can improve some outcomes 17](#_Toc54550356)

[7.3 Stories are acceptable and engaging 17](#_Toc54550357)

[7.4. Story-based online games 17](#_Toc54550358)

[8. Considering the needs of specific population groups 18](#_Toc54550359)

[8.1 Summary of evidence: 18](#_Toc54550360)

[8.2 Cultural diversity 18](#_Toc54550361)

[8.3 People living with a disability 19](#_Toc54550362)

[8.4 People with lower health literacy or lower education 19](#_Toc54550363)

[9. Empowerment/Patient Activation/ /Self-Efficacy 19](#_Toc54550364)

[9.1 Summary of evidence: 19](#_Toc54550365)

[9.2 Empowerment and the role of communication 20](#_Toc54550366)

[9.3 Patient activation 20](#_Toc54550367)

[9.4 Self-efficacy 20](#_Toc54550368)

[10. Use of theories or frameworks 21](#_Toc54550369)

[RECOMMENDATIONS FOR THE CONSUMER-LED TEACH-BACK ONLINE RESOURCE 21](#_Toc54550370)

[REFERENCES 23](#_Toc54550371)

**Consumer Check-back: A rapid literature review to inform development of an online learning resource for consumers**

# BACKGROUND

Effective patient-provider communication is an essential component of good health care. A significant body of evidence demonstrates that high quality patient-provider communication is associated with many positive benefits, whereas poor communication is shown to lead to adverse health outcomes and increased healthcare costs (1-7) (8). Communication between patient and provider is thought to have an indirect effect on health through its influence on patient understanding, clinician–patient agreement on treatment, and adherence to treatment (9) (10).

Evidence indicates that lack of understanding by patients is common, with one study identifying that 37% of patients did not understand the reason for their hospital admission (11). Patients may also not understand as much as the clinician thinks they do (12). In one study, 77% of doctors believed patients knew their diagnosis; however, only 57% of patients could correctly state this (13). Many patients also have difficulty remembering information from their medical consultations, which can negatively impact their understanding of their condition and adherence to treatment (14). In one Canadian study, patients recalled only 50% of lifestyle or medication discussion (15)***.***

One communication tool shown to lead to improved consumer understanding and recall across diverse settings and health conditions is teach-back (16-18) (19). Teach-back involves clinicians using plain language to explain health concepts, and then asking the consumer to explain back this information using their own words. Strong evidence indicates that use of teach-back can lead to improved knowledge, skills and self-care abilities in consumers (20) (21) (22). In a recent systematic review, teach-back was shown to be effective in 19 of 20 included studies. Studies were conducted across a wide variety of settings including hospitals, emergency departments, outpatient clinics, primary care practices, community health centres and nursing homes. Participant groups included children, older adults and younger adults, people with chronic conditions, post-surgical inpatients, new mothers, and people discharged from hospital (22).

Teach-back is a particularly useful tool in healthcare, as it can be applied universally with all consumers independent of their level of health literacy. This means that clinicians do not need to have an understanding of a consumer’s health literacy abilities. For this reason, teach-back is now recommended in many policy documents and position statements, including the Australian Commission on Safety and Quality and the Agency for Healthcare Research and Quality (23, 24).

Use of teach-back builds greater rapport and more active communication between patients and clinicians (25), and if used respectfully may help reduce any stigma or perception on the part of the consumer that it is their ‘fault’ they do not understand or are unable to work something out (26, 27). Yet despite the fact that teach-back offers a simple and effective approach for improving healthcare communication, the method has had minimal uptake to date (28). Many health professionals do not routinely check whether patients understand the information they provide, nor do they explore what the patient already knows and what information is still needed (29) (15). A survey of US dentists showed that fewer than one-fourth of respondents reported using teach-back (30).

A possible explanation for the low uptake of teach-back is a perception that it increases the length of the appointment (28, 31-34) (35), yet evidence shows that using the teach-back method adds just 2.6 minutes to dental visits (36). Clinicians may also feel uncomfortable with phrasing, or that they may appear to be condescending (37, 38). Some clinicians have also shared that teach-back can be cumbersome to use and that they needed reminders to use it consistently (38, 39). However, when asked, patients are generally interested in the idea of teach-back, reporting that it would help them confirm their learning and support them to remember important information (25, 26). One group of parents in the US suggested that use of teach-back demonstrates caring by the provider, and recommended that consumers should ask for it to be used (25).

Given this general acceptance among consumers, teach-back has the potential to become more of a consumer-led rather than a clinician-led approach. For example, by consumers saying to a clinician: “Let me make sure I understand – Each morning, I should……”. Use of consumer-led teach-back may increase consumer participation and engagement as well as increasing their understanding of what they need to do. However, while communication skills such as asking questions and giving information may come naturally to many patients, initiating teach-back requires more advanced skills (40). These include the ability to actively listen, to summarise information, to inform the clinician they are about to use teach-back, and then to repeat back key information (40).

Although there are many available resources about how to talk with doctors or clinicians, such as Better Health Channel, few of these include advice about the importance of checking information through use of the teach-back technique. Further, very few studies have investigated whether it is feasible or effective to educate consumers to initiate teach-back. This evidence brief was undertaken to inform development of an online learning resource to educate consumers about the use of teach-back. The resource aims to support consumers to see the importance and value of checking their understanding, to provide them with the necessary skills and confidence to initiate teach-back themselves in any interaction with a healthcare professional.

# AIM

To inform the content and delivery of an online learning resource for consumers to initiate teach-back themselves through a rapid review of the academic literature about communication skills training for patients.

# METHODS

Rapid literature reviews are a way of synthesising knowledge that omit some elements of a traditional systematic review in order to simplify the process. Rapid reviews and systematic reviews are considered to be generally congruent in their findings (41).

## Search strategy

To address the aim of this review, we searched the academic database *Ovid Medline* and reference lists of retrieved articles. The search was driven by the following research questions:

1. What strategies directed at patients are effective in improving patient-provider communication?
2. What are the core elements of effective online learning strategies for consumers that aim to improve patient-provider communication?
3. What are the core elements of effective patient empowerment strategies that aim to improve patient-provider communication?

### Inclusion criteria

- 2011-2020. English Language
- Study design - observational or exploratory studies (non-interventional), intervention studies (RCTs, Pre/Post, Case Control etc.), methodology can be quantitative or qualitative.
- Studies that report a strategy or intervention aiming to improve patient-provider communication. The intervention must be targeted towards the patient/consumer and may aim to directly improve patient-provider communication or to change patient health outcomes, behavioural outcomes, or health literacy through improved communication.
- Or studies that report a strategy or intervention aiming to increase patient/consumer empowerment, autonomy, or self-efficacy in relation to communication with providers.
- Or a systematic review of studies reporting online education or web-based learning or education interventions directed at patients/consumers, and that aim to change patient/consumer knowledge or understanding, or health and behavioural outcomes.

### Exclusion criteria

Studies directed at carers or health professionals (unless delivered via the health professional).

## Data extraction and analysis

Titles and abstracts were reviewed and studies not considered to meet the above inclusion criteria were excluded. The remaining full texts were then reviewed for inclusion by two authors. During full text review, we identified key themes across the literature. Studies were then reviewed and data extracted according to each key theme. A narrative approach was used to report the findings.

# RESULTS

In total, 66 studies were included; 20 of these were systematic or scoping reviews. Figure 1 shows the number of studies that were excluded.

| **Figure 1: Results of literature search** | | | | |
| --- | --- | --- | --- | --- |
|  |  | | |  |
|  | Number meeting inclusion criteria on Medline search =1,247 | | |  |
|  |  |  |  |  |
|  | Excluded on title and abstract review = 943 | | |  |
|  |  |  |  |  |
|  | Excluded on full text review = 227 | | |  |
|  |  |  |  |  |
|  | Additional studies identified through reference lists = 11 | | |  |
|  |  |  |  |  |
|  | Final number of studies included = 66 | | |  |

Of the 46 studies that were not systematic reviews, the majority were conducted in the USA or Canada. There were two studies from Australia. Patient groups ranged from parents of children or infants, adolescents to older people, those with diverse cultural or socioeconomic backgrounds, or those with lower health literacy. Health conditions included cancer, mental health conditions, heart failure, and preventive cancer screening. Settings included hospitals, community health centres, workplaces, or medical clinics.

Key themes are shown in Table 1 and discussed further below.

| **Table 1: Key themes used for data extraction and reporting** |
| --- |
| 1. Patient-provider communication |
| 1. Skills training for communication |
| 1. Computer aided learning (CAL) in healthcare |
| 1. Design & delivery elements of CAL |
| 1. Value of adding printed resources |
| 1. Visual communication tools |
| 1. Use of stories or narratives to deliver messages |
| 1. Tailoring to population groups |
| 1. Empowerment/patient activation/self-efficacy |
| 1. Frameworks/theories |

## 1. Patient-Provider Communication

1.1 Summary of evidence: There are many influences on patient-provider communication, including differences in skills and demographic characteristics between the patient and clinician. Patient-reported barriers include a perceived power imbalance, fear of clinicians, the clinicians’ communication style, emotions, beliefs or lack of confidence about their own conversational skills or cognitive abilities. Patients’ level of participation is also a strong influence on clinicians’ communication. Most of the evidence about patient-provider communication comes from the clinician perspective, and there are calls for more patients’ perspectives. Some authors suggest that interventions to improve patient-provider communication should focus on creating patients’ awareness about the role they can play and on training them in communication skills.

### 1.2 Influences and barriers to effective patient-provider communication

In the US, surveys of 306 patients identified that influences on patient–provider communication included differences in communication skills, age, racial, ethnic and linguistic background, gender differences, and differences in educational background and socioeconomic status (42). One influence on provider behaviour is the patients’ level of participation. A systematic review found that providers offer more information and make more partnership-building statements to patients who ask questions, or otherwise communicate actively in visits (40).

Barriers to interaction can include a perceived power imbalance between patient and clinician such as a patient’s perception that they have limited expertise compared to the physician’s knowledge (43) (44). The communication style of some healthcare professionals may also hinder effective communication, for example by being hasty or by communicating in an overly technical way (44). Patients may also perceive several barriers within themselves, such as being overwhelmed by emotions, their own values and beliefs, and feeling they lack the conversational skills or cognitive abilities to actively participate in healthcare visits (44).

Fear of physicians (‘iatraphobia’ ) is a common phenomenon and can lead to less confidence in the quality of healthcare and less patient autonomy, avoidance of physicians and greater use of informal source of healthcare (42). One study assessed associations between patients’ fear of physicians and their perceptions of physicians’ cultural competence in healthcare interactions finding that fear of doctors was less when there was greater patient-centeredness during the medical encounter (42).

### 1.3. Importance of including consumer/patient perspectives on communication

Most evidence about patient–provider interactions is from the perspectives of healthcare providers and there are calls for greater involvement of patients’ perspectives on patient–provider interactions (44, 45). A 2017 systematic review about training patients in the use of effective communication skills found that the overwhelming majority of work in healthcare communication has centred on the providers’ side of the clinical encounter, including interventions to improve providers’ communication skills (40). While most medical training programs have addressed physicians’ communication skills, few programs or projects have addressed the communication skills of the patients or focused on the patient’s contribution to communication (46).

In The Netherlands, interviews with 29 patients revealed three transitional states in communication with healthcare professionals: 1) being overwhelmed and passive, 2) being pro-active and 3) being self-motivated, proficient and empowered. The authors suggest that in line with the ‘conscious competence learning’ model by Maslow, moving towards a next state in this trajectory might be facilitated by increasing patients’ awareness on the role patients can play and the benefits of active participation. The authors suggest that interventions should focus on creating awareness about the role patients can play during consultations and on training in communication skills (47).

## 2. Skills Training For Patient-Provider Communication

2.1 Summary of evidence: Evidence for the effectiveness of training patients in communication skills is mixed. Some systematic reviews and other studies identify that communication training can improve patient participation and understanding, partnerships and patient-centred communication. Other studies have found that there is limited evidence for its effectiveness in improving health outcomes, medication adherence or patient-doctor relationships. Most of the interventions were paper-based or delivered face-to-face. Some included ‘patient rehearsals’ or role play, where patients could practice communication and question-asking. One study included training for patients about the importance of checking their understanding, delivered within a face-to-face communication workshop.

### 2.2 Interventions delivered to patients only

A systematic review of 38 studies on patient communication interventions found that communication training or coaching is an effective approach to increase patients’ active participation and the amount of information they receive from their providers. However, most studies found no relationship between communication training and improved health or psychosocial wellbeing. Of the included studies, 41% were materials-based rather than interactive, including the use of workbooks, leaflets or multimedia. Eight studies looked at ‘checking understanding’ skills, with half finding a difference in favour of training and half finding no differences (40).

Another study assessed a one-on-one peer support intervention for 98 patients newly diagnosed with HIV. Participants received training in communication strategies including role play about how to give and receive information effectively. Patients were taught that it is appropriate to ask questions, and were given specific examples and scenarios in which questions might arise. Participants in the intervention fared significantly better regarding illness uncertainty and depression, but not self-advocacy (48).

In a RCT of a patient-driven communication intervention, patients were given a structured communication tool developed by physicians. Patients were given a form to complete before their appointment which included why they wanted to see the doctor, any recent changes or improvements, and any current issues. Patients who completed the written form reported better doctor-patient communication skills in understanding their health, communicating their concerns, and feeling that they were partners with their physician (46).

A pilot study conducted among a minority cancer patient population examined the effectiveness of a communication workshop to improve doctor-patient communication skills. Thirty-two patients participated. Each workshop consisted of a 20-30 minute PowerPoint presentation, video clips demonstrating the skills taught in the workshop, and discussion. The intervention included a section on checking understanding. Participants in the workshop group all agreed that they would use the communication skills, with 93% agreeing that the skills would improve their healthcare (49).

A systematic review of patient coaching interventions found significant improvement on immediate, intermediate and long term patient-physician communication. The authors found some evidence suggesting an improvement of patient-physician communication by having multiple patient coaching encounters during which questions are prepared and rehearsed (44).

### 2.3 Interventions delivered to both patients and clinicians

In a RCT in Baltimore, researchers conducted physician communication skills training and patient coaching by community health workers over one visit. Patients practiced disclosing concerns, asking questions, and stating preferences; and were helped to identify strategies to overcome anticipated problems. The intervention also included a physician-training program including feedback on their videotaped performance with a simulated patient. The authors found no differences in patient medication adherence between the intervention and control group (50).

Another RCT evaluated whether a combined intervention involving oncologists, patients with cancer, and caregivers would promote patient-centred communication. Oncologists received individualised communication training using standardised patient instructors while patients received question prompt lists and individualised communication coaching. The study found improvement in the primary outcome of patient-centred communication, but not in the secondary outcome of improved patient-physician relationships (51).

## 3. Computer Aided Learning in Healthcare

3.1 Summary of evidence: Computer aided learning (CAL) – any learning that is mediated by a computer – can have a positive impact on treatment adherence and skill development, and is show to be as effective among people with lower health literacy. Only one study was identified that used CAL specifically for communication training, finding the intervention was associated with meeting treatment targets in patients with chronic disease. This intervention included training for patients about the importance of checking their understanding.

### 3.2 What is Computer Aided Learning (CAL)?

Computer aided learning (CAL) is defined as: “any learning that is mediated by a computer and which requires no direct interaction between the user and a human instructor in order to run” (52) CAL can be enhanced with audio and video components, and can also include interactive elements such as feedback, discussions, forums, and quizzes. While costly to set up, CAL usually requires less time and fewer resources than traditional didactic methods (52).

### 3.3 Is CAL effective for patient learning?

Computer or internet-based education has been shown in a number of studies to have a positive impact on clinical outcomes, skill development and self-care management (53-57). A systematic review of treatment adherence interventions in dermatology found four studies investigating the use of the internet and audio-visual techniques to encourage adherence, all reporting a significantly positive effect (54). The review included a study testing adherence to sunscreen use which demonstrated that audio-visual instruction (online video) was more effective for conceptual learning than written materials, highlighting the importance of the vehicle used to deliver the message (54). Another systematic review of Internet and computer-based education programs for patients with prostate cancer found that these can be useful tools but study methodology is of variable quality, indicating that more robust research is required (53). CAL also appears to be generally acceptable to consumers (56).

CAL is also effective among people with low health literacy. A review of eHealth interventions to improve consumers’ health literacy found that computer-based applications were the most common delivery platform and were associated with significant positive changes in health outcomes and/or health literacy scores. The authors concluded that it is feasible to deliver eHealth interventions to improve health literacy for people with different health conditions, risk factors, and socioeconomic backgrounds. The interventions targeted a variety of health risks, lifestyles, and disease management and all included at least one health literacy component (55).

### 3.4 Is CAL effective for communication training?

An RCT in a community primary care setting in Canada investigated the impact of a web-based communication intervention for 322 patients with chronic disease. The e-Learning intervention guided patients through each of the four sections of the PACE approach (Prepare, Ask, Check, Express) to improve physician–patient communication and patient participation. Patients were able to enter information to be printed for their use. The website presented content via audio, stand-alone text, narrated text and representations of different doctor-patient encounters. The ‘Prepare’ section focused on helping patients organize information to share with their physician. ‘Ask’ encouraged patients to think about questions and write them down. ‘Check’ encouraged patients to verify and summarize their understanding. ‘Express’ encouraged patients to voice their concerns. Patients in the web group were 1.42 times more likely to meet targets compared to usual care indicating that a web-based communication intervention using PACE positively impacts treatment targets for patients with chronic disease (58).

## 4. Recommended Design & Delivery Elements of CAL

4.1 Summary of evidence: Using mixed modalities for delivery of information is effective, including illustrations, videos, text, animation and narration within the one CAL program. Websites should use large clear font, brief and jargon-free information, consistent layouts, and links to external websites. Developing and testing materials with the target population is also recommended. Generally, online educational interventions which include an interactive component are more effective than those that are less interactive. Allowing participants to jump in and out of the module is also recommended.

### 4.2 Delivery modes and layout

In a systematic review of eHealth interventions to improve health literacy, authors found that many Computer Aided Learning (CAL) interventions used a mix of modalities for delivering content, e.g. narrated PowerPoint presentations, illustrations, 3D animations, photos, text, and narration within the one intervention (55). The use of multiple modes of delivery may also address the diversity of learning styles found within any population (59)

It is recommended that websites should use a large, clear font size (e.g. Arial 16) and high contrast lighting to allow text to stand out. Other characteristics include links that are distinguishable from texts, readable language, consistent layouts across the various screens to facilitate ease of navigation, use of a mouse or keyboard to select links, and a talking browser (60)

A systematic review of information provision in palliative care for patients with low health literacy recommended key strategies in delivering online information as: a) Providing clear, brief, jargon-free information in a conversation style (active voice), supported by graphs, illustrations or visuals; b) Using large font size and ample white space; c) Developing and testing materials with the help of members of the target population; d) Using short sentences and paragraphs; e) Using audio and video recordings as presentation materials (29).

A systematic review of internet and computer based programs for prostate cancer patients found that graphs, priority information topics and annotated external website links were reported as most useful (53).

### 4.3 Importance of interactivity

In a systematic review of interventions to improve patient comprehension in clinical informed consent, the authors found that interactive digital learning interventions improved patient understanding, even more so for those with test/feedback or teach-back components (61). Findings from the review suggest that interactive informed consent interventions (i.e., those that intentionally promote active patient involvement), may be superior to non-interactive interventions (61). A review of web-based interventions targeting mental health literacy similarly found those that include active ingredients are more likely to be successful (57). Another systematic review of 19 studies aimed to determine the most effective components of web-based education interventions for self-care behaviour. Findings suggested that the most effective form is one that is interactive and allows patients to navigate the online system on their own (60).

An example of interactivity is a U.S. study that evaluated a web-based tool to help patients with schizophrenia communicate with clinicians. Patients used an interactive web-based program featuring actors simulating a patient discussing treatment concerns (intervention group; n=24). Within the intervention, patients answered questions about their current status and treatment. On the basis of their responses, individualized feedback recommendations appeared on the screen. Compared with the control group, visits by patients receiving the intervention were longer and had a greater patient contribution to the dialogue. Patients asked more questions about treatment and more often checked that they understood information. The patient-centeredness ratio was greater for visits by patients in the intervention group than by the control group (62).

Use of online gaming is another form of interactivity. An mHealth tool was developed as an online game to support patients with lung cancer with navigation of their cancer journey. Patients as ‘‘players’’ arrive at a bus stop outside a virtual cancer centre, where they are met by a ‘‘coach’’ to offer explanation and support throughout the experience. Players experience successful navigation of a clinic visit through increasingly complex situations, giving them practice addressing issues and asking questions, which thereby develops skills they can use in their real world clinic visits. Users saw the goal of the game as preparation for ‘‘me [a player] to be an informed consumer of healthcare’’ (63).

### 4.4 Additional considerations in design

Metaphors can be an effective health communication tool and have been used as a strategy to provide a common language for clinicians and patients, including successfully explaining molecular testing to cancer patients (64). Metaphors are also effective among people with lower health literacy. One study used metaphors to explain randomisation (for participation in cancer randomised controlled trials) among patients with diverse levels of health literacy. The authors found that a benign metaphor was particularly effective for patients at the lower end of the health literacy spectrum (the metaphor compared randomisation with the chance of have a baby boy or girl) (65).

In terms of their viewing flexibility, allowing participants to jump in and out of the module may be effective. A systematic review of computer-aided learning in oral health identified that flexibility to view the program without any restriction in timing leads to more positive outcomes, including increased satisfaction and concentration. In turn, this may lead to higher compliance and usage of CAL programs (52).

## 5. Value of Adding Printed Resources

5.1 Summary of evidence: Question prompt lists (QPLs) are a structured list of questions provided to patients, who are then encouraged to ask relevant questions during consultations with physicians. QPLs aim to increase patient participation in consultations, improve patient knowledge, and promote information exchange. Overall, systematic reviews and other studies have found that QPLs are effective, particularly for people with cancer but there are some caveats around their accessibility and if not endorsed by clinicians, they may increase patient anxiety. Frequently Asked Question (FAQ) sheets or fact sheets can be useful for patients, especially if they are visually appealing and include colours and images.

### 5.2 Question Prompt lists – should these be included as an additional resource?

In a review of systematic reviews about communication tools in cancer consultations, it was found that patients using QPLs asked more questions during consultations. The included reviews found no difference in terms of patient satisfaction, and there were mixed to equivocal results in terms of patient anxiety. Overall, there was no effect of QPL use on depression, psychological adjustment, or distress. However, there was a positive effect on recall of information and the number of questions asked, with no significant increase in consultation time (66). In a review of interventions of patient-professional communication in life-limiting conditions it was found that enabling patients to ask questions may lead to improvements in communication. Using a QPL 20 minutes before a consultation, showed a discussion of more issues and an increase in the number of prognostic questions asked by the patients, leading to reduced uncertainty about what to expect in the future (67). QPLs are also effective among people from culturally diverse or lower socioeconomic groups. In a rural, underserved setting in the U.S., listing questions helped patients prepare for oncology visits, leading to higher self-efficacy and lower anxiety (68). Among culturally diverse and low English-speaking patients with cancer in Melbourne, the QPL was described as being useful, particularly to prompt questions they had forgotten or not considered. Some patients thought that QPLs should be available to all patients upon arrival, and that it would be particularly useful for people with lower education levels, or who were newly diagnosed (69). Barriers to their use include potential lack of endorsement from clinicians leading to increased anxiety among patients (66). Interviews with clinicians and hospital administrators in the U.S. also identified potential issues related to their relevance and accessibility and uptake (70).

QPLs can be of varying length and complexity. In a Swedish study a QPL was co-designed with patients and carers to facilitate patient-clinician communication about heart failure. This QPL was a 7-page booklet, containing 45 questions grouped into 5 topics (71). In Australia, the [**Choosing Wisely**](https://www.choosingwisely.org.au/resources/consumers-and-carers/5questions) initiative includes just five questions to determine the benefit and needs for tests or treatments. The questions are: 1) Do I really need this test, treatment or procedure? 2) What are the risks? 3) Are there simpler, safer options? 4) What happens if I don't do anything? 5) What are the costs? The strategy has been evaluated within Australia where it was found that among people with lower health literacy, exposure to the Choosing Wisely Australia® 5 questions was associated with an increased intention to engage in shared decisions about low-value care (72).

### 5.3 Fact sheets/Frequently Asked Question (FAQ) sheets

In the US, focus groups were conducted with 20 vaccine-hesitant parents to understand how a multi-faceted communication intervention could improve uptake of Human Papillomavirus (HPV) vaccine. The intervention included a fact sheet library and a parent website that created individually customized information. Parents consistently selected the fact sheet as a favourite tool in the intervention citing that it was easy to understand, answered common questions about HPV, and was visually appealing and engaging with colours, figures and images, FAQs were also reported as being very useful. Comments were that the “Fact sheet answered a lot of questions and you could start a conversation with your doctor if you hadn’t thought of those things. The graphics and the colour and how it pops and grabs your eye” (73).

## 6. Visual Communication Tools

6.1 Summary of evidence: Web-based videos are effective tools to demonstrate practical skills, model behaviours, and provide advice and education. Pictorial tools such as infographics, comics and images are generally well received by patients, and may be particular useful for people with lower health literacy, cognitive challenges, or those who do not speak the local language. Infographics that are “information-rich” are often more easily understood, and the use of colour to denote meaning is also recommended. Care must be taken not to trivialise patient situations through stereotypical pictures or cartoons. Graphs can also be a powerful way to convey health-related statistical information, especially for those who have problems with understanding and applying numbers. However, caution is needed as graphs may not be intuitively understood by everyone.

### 6.2 Value of videos to model behaviours and provide information

One author evaluated whether a web-based program could empower patients with schizophrenia to discuss quality of care with mental health providers. The program includes 14 x 30-second video clips designed to model the performance of communication strategies and skills using actors as simulated patients. The clips provide examples of how someone with schizophrenia can broach potentially sensitive issues with a therapist. Communication strategies to assist the patient to be proactive were also modelled in video clips, including setting the visit agenda by expressing goals, using paraphrasing to check for understanding, asking for plain words instead of scientific terms, and summarizing important information at the close of the visit. Patients exposed to the intervention were more verbally active during mental health visits than control group patients (62).

### 6.3 Infographics or pictograms

Concrete tools such as pictographs have been used to simplify complex medical concepts, and are used to communicate instructions for medication use (e.g., time of day, with or without food) among elderly and low literacy patients (74). Pictorial tools can be used online or in printed form and are particularly important among populations with minimal education, challenged cognitive skills, or in settings where providers are not fluent in the varied languages or dialects of their patients (74). In the U.S. one author evaluated pictorial images to communicate the importance of medication timing and drug resistance in Antiretroviral therapy. The images were both positively received and effective in increasing medication knowledge among a diverse population (74).

Visualizations are thought to work best when pictures illustrating key points and simple text are closely linked and potentially distracting and irrelevant details are omitted (75). In the U.S. infographics for health promotion were co-designed with community members. The most easily understood infographics were those that were information-rich, provided context, and/or employed familiar analogies. Participants frequently emphasized that “more information is better.” Participants responded well to symbolic analogies, and colour meanings were well understood (e.g. red for danger). Some images were interpreted too literally. For instance, participants who were shown multiple apple icons instead of fruit cluster icons complained about the monotony of eating the same fruit every day (75).

Colours were also discussed in another study assessing the validity of a tablemat for involving hospitalised patients and carers in their care. Comments included making the colours more contrasting, including darker lettering, and emphasising key messages in separate red squares. Using affirmative slogans/messages and more active voices were also recommended (76).

In Japan, one study explored the use of medical comics to obtain informed consent. The authors surveyed patients, finding that medical comics may facilitate informed consent during emergency conditions, such as strokes, as they encourage the patients and their families to understand the facts, implications, and future consequences of an action within a short period of time (77)

In the US, an exploratory study with 18 children and 21 caregivers explored weight-related communication for children with disabilities. Findings identified that visual tools using cartoon characters were deemed offensive and unhelpful by most caregivers and older youth, especially those using stereotypical pictures (e.g., junk food) to depict characters with larger bodies. Younger children did see some humour in them that could potentially be engaging, but older youth did not want their growth information trivialized. One participant (aged 15 years) explained: “If it's a serious topic, just tell it to me like it's serious, don't … put this hocus pocus drawing stuff in front of me” (78)

### 6.4 Graphs & numbers

Graphs can be a powerful way to convey health-related statistical information, especially for those who have problems with understanding and applying numbers. However, caution is needed as graphs may not be intuitively understood by everyone (79, 80). People have different levels of proficiency in understanding graphs and numbers, even among people with higher levels of education. One study assessed comprehension of quality of life dashboards among prostate cancer patients, 78% of whom were college educated. The study found considerable variation in the ability to comprehend graphs. The authors note that tools for patient-centred communication using visual displays need to account for literacy capabilities to ensure that patients can effectively engage these resources (79). A review of current “best practices” in communication of numbers highlights the need to use a consistent format throughout, to consider the magnitude of numbers used, and should take into account the numeracy and graph literacy of the audience.

## 7. Use of stories or narratives to deliver messages

7.1 Summary of evidence: Stories may be effective at delivering information because they are easy to understand and more likely to be remembered. An appeal of storytelling is its ability to present information that engages the reader and validates their own experiences (81). People become absorbed in the plot, and may be less likely to reject (or even notice) counter-attitudinal information. Stories provide opportunities to depict characters engaged in the target behaviour or overcoming barriers to action, and this use of modelling can increase self-efficacy (82). Narrative communication can thus be viewed as a form of learning through experience (83). Overall, the evidence suggests that use of stories to deliver messages is engaging for participants but there is limited evidence for their effectiveness, and they may need to be supported by other strategies. Stories have been delivered via written pamphlets, photo story booklets, videos, web-based messages. Only one study developed a booklet to support better patient/provider communication, this was not evaluated for its effectiveness but was appealing to participants, especially those with lower health literacy (84). Use of an online ‘personal guide’ to provide a narrative was shown to help patients with cancer feel like informed consumers (63).

### 7.2. Stories and narratives can improve some outcomes

Narrative messages have been used to increase uptake of cancer screening. These narratives can depict individuals grieving lost loved ones, overcoming challenges, or using services. In a workplace intervention to improve colonoscopy screening, individuals receiving narrative messages were 4 times more likely to screen than those not receiving narrative messages (82). Stories were tailored to ethnicity and gender and delivered as messages via the web and could also be printed out as pamphlets.

In the US, authors developed a cancer-communication intervention for African American breast cancer patients using cancer survivor stories. The authors included 207 clips told by 35 survivors in an interactive video program shown on a touch-screen computer. Videos could be searched by storyteller or story topic. Evaluation with 10 survivors found that stories were “interesting and informative,” and usability was rated highly. Participants identified with storytellers (e.g., they “think a lot like me,” “have values like mine”) and agreed that the stories convinced them to receive recommended surveillance mammograms (85).

In a Canadian study, written stories were provided as booklets about parent experiences managing a child with croup. Booklets did not reduce parental anxiety, however the story group showed significantly quicker time to resolution of symptoms. The purpose of the stories was to deliver health information in the context of real parent experiences (81).

A similar format for delivery is the fotonovela. These are small booklets, similar to comic-book formats, with photographs instead of illustrations, combined with dialogue bubbles or captions. They are designed to engage audiences with realistic characters, simple texts, and vivid pictures (84). When used with patients with diabetes, participants showed significantly higher increases in knowledge, with this finding seen consistently across literacy levels. On behavioural intentions, however, readers of the fotonovela did not score significantly higher than participants in the other conditions (84).

### 7.3 Stories are acceptable and engaging

In a European study, authors compared a narrative photo story booklet about doctor-patient communication with a non-narrative but otherwise highly similar brochure. The photo story booklet included seven short picture-based stories about themes related to doctor-patient communication. The non-narrative brochure had comparable pictures and layout and dealt with the same themes, but did not include any stories. Authors conducted two RCTs among older adults with varying levels of health literacy. There were no significant differences between the photo story booklet and the non-narrative brochure; however most participants expressed a preference for the photo story booklet, which was perceived as relevant and engaging, particularly for people with lower health literacy (83).

### 7.4. Story-based online games

An mHealth tool was developed as an online story-based game to support patients with lung cancer with navigation of their cancer journey. Patients as ‘‘players’’ arrive outside a virtual cancer centre, where they are met by a ‘‘coach’’ who presents themselves as a knowledgeable friend (neither patient nor provider) who offers explanation and support throughout the experience. The coach provides a narrative (i.e., story of a patient who previously visited this virtual clinic and achieved optimal health literacy and self-management). Players experience successful navigation of a clinic visit through increasingly complex situations, which thereby develops skills they can use in their real world clinic visits. Users saw the game as believable, for example, they reacted strongly when confronted with ‘‘unhelpful’’ clinic staff – evidence of the game’s ability to emotionally engage players. Users saw the goal of the game as preparation for ‘‘me [a player] to be an informed consumer of healthcare.’’ (63)

## 8. Considering the needs of specific population groups

8.1 Summary of evidence: Overall, there is limited evidence available for the effectiveness of tailoring communication resources to the needs of under-represented population groups. For culturally diverse groups, linguistic translation is not enough, cultural values and norms must also be considered. Including both English and the language version is suggested to allow for people who use blended language patterns. Visual aids and showing people from the same cultural group are also recommended. Cultural beliefs to consider are discomfort with talking to providers and asking questions, or “wasting” their time. People with lower education or older people need more support for being able to use eHealth; a suggestion is to include tutorials, and inclusion of family members may also be important (for example in videos). For people living with a disability, one study among first-time adult hearing aid users recommended the use of images and animations plus a transcript of any audio or video commentary. For people with lower health literacy, eHealth interventions are shown to be effective. Clear communication strategies are recommended, regardless of health literacy ability.

### 8.2 Cultural diversity

Research has shown that in the US, ethnic and racial minorities receive suboptimal health communication compared to white patients (40). One study examined engagement, confidence, and communication among Latinos and non-Latinos. In age-adjusted models, Latinos were significantly less likely to report knowing how to ask good questions about their health (86). In another study among Spanish-speaking patients in the US, not having a clear understanding of medication regimens led to frequently missed doses. Tied into this were cultural variations in attitudes toward health, specifically health or folk beliefs (87).

Tailoring to particular cultural groups is warranted; however, this involves more than linguistic translation. Simple translation will not address cultural values and norms (88). A review of eHealth among Latino populations recommended integrating both English and Spanish languages when designing health messages. This style of message is particularly important with younger populations because it mirrors their blended language pattern. Latinos who spoke limited English also appreciated health messages that included visual aids, images of Latino-appearing people, and simple language more so than those with more refined English, who wanted more information (89).

In one study of a communication intervention comprising consultation audio‐recordings and question prompt lists (QPL) for low English‐speaking patients with cancer, participants reported that the resources supported them to remember and understand medical information. However, while many patients felt that the QPL was useful, several felt uncomfortable asking questions as they did not want to “waste” the interpreters' or doctors' time. Other participants felt that only doctors, not patients, should ask questions. Some patients thought that it was more appropriate to rely on family to prompt question‐asking, rather than the QPL. However, the translation and cultural acceptability of the QPL was reported to be good (69).

### 8.3 People living with a disability

Only one relevant study was identified. This was a UK study aiming to develop content for a series of interactive video tutorials for first-time adult hearing aid users, guided by recommendations from an Expert Panel. Recommended components included use of learning goals, video clips, animations, still images, a transcript of the text to accompany the media (both audio commentary and subtitles), and an interactive multiple-choice quiz with feedback. Multiple short videos were considered to be more usable and effective than a single video covering multiple topics. The Expert Panel also identified that video content should be informal and patient-focused. Other elements included reinforcing the importance of practicing new communication skills (90).

### 8.4 People with lower health literacy or lower education

People with lower health literacy are more likely to report poor communication in terms of general clarity and explanations (59). A systematic review of information provision in palliative care for people with low health literacy, found that patients ask fewer questions and take less control, but nevertheless do wish to take part in decision-making as much as other patients (29).

A systematic review and meta-analysis of self-management interventions for people with lower health literacy and diabetes identified a range of effective strategies. Five studies used a “clear communication” strategy, such as using common words, limiting content to 3–5 key points, repeating key points, and drawing pictures when speaking to patients. Four studies used the teach-back method to ensure that participants understood diabetes education. Interventions incorporating spoken communication were typically used in conjunction with other strategies (91).

A UK study interviewed nine older adults attending a falls clinic. Participants expressed clear preferences for tailoring of written information to their individual needs and attributes. Regardless of their health literacy level, participants valued information being delivered in a clear and simple manner (59).

A systematic review of 12 eHealth interventions for low health literacy found that overall, it is feasible to deliver eHealth interventions specifically designed to improve health literacy skills for people with different health conditions, risk factors, and socioeconomic backgrounds. Computer-based applications were the most common intervention delivery platform; Several used a variety of modalities for delivering content. Improvements were seen in knowledge and HIV testing; fruit and vegetable consumption among adolescents; colorectal cancer knowledge scores, perceived risk scores, and colonoscopy benefit scores; disordered eating, and excessive exercise (55).

In a systematic review of sociodemographic factors influencing the use of eHealth; lower income, lower education, older age, and living in rural areas were associated with lower eHealth use. To reduce the “digital divide”, it is recommended that people with less education and older people need additional support when using eHealth. One suggestion is to include tutorials in the eHealth application. Examples in which people can better recognize themselves should be provided. Inclusion of family members may also be important (for example in videos modelling behaviours) (92).

## 9. Empowerment/Patient Activation/Self-Efficacy

9.1 Summary of evidence: Patient empowerment can be influenced by the way in which health providers communicate. One systematic review found that eHealth programs including video clips enhanced patient empowerment to communicate with their provider. Other studies did not specifically focus on communication, although one study used peer champion stories to build self-efficacy. Interventions can improve patient-doctor communication by improving patient activation, and using adult learning principles to improve their self-efficacy and conversation skills.

### 9.2 Empowerment and the role of communication

Empowerment is a concept that was introduced to allow patients to play an active part in decision-making about their health. A proposed definition of patient empowerment is: A process that enables patients to exert influence over their health by increasing their capacity to gain more control over issues they themselves define as important (93). Patient empowerment can be influenced by the way in which health providers communicate. In a study of renal physicians’ communication, ‘empowerment failure’ occurred because of failure to communicate clearly. The urologists in this study acknowledged the importance of their role as information providers, but did not have the ability to explain medical concepts in ways that most patients could understand (27).

In a systematic review of internet and computer-based programs for prostate cancer patients, nine of the included interventions incorporated video clips instructing patients on how to speak about personal matters with their doctor. The majority of these studies reported a significant increase in disease knowledge, satisfaction with treatment options and support for men. Patients were more empowered to discuss their disease and were thus able to control and deal with their condition (53).

In a systematic review of web-based interventions for patient empowerment and physical activity in chronic disease, the authors found significant, positive effects on patient empowerment in just four of 18 included studies (94).

One study aimed to build patient empowerment in order to reduce inappropriate benzodiazepine prescriptions among older adults. The intervention consisted of an 8-page booklet based on self-efficacy theory, and included a self-assessment component about the harms of benzodiazepine use, and peer champion stories intended to augment self-efficacy. At 6 months, 27% of the intervention group had discontinued benzodiazepine use compared with 5% of the control group (95).

### 9.3 Patient activation

Patient activation can be defined as having the knowledge, skills, and confidence to manage one's health, and is shown to be strongly associated with a broad range of related to health-related outcomes (96).

One US study sought to evaluate the impact of a patient activation intervention focused on building question-asking skills among low-income, racial/ethnic minority patients in community health centres. In the intervention, researchers helped the patient understand their decision-making processes, brainstorm questions that might be helpful during an up-coming visit, and understand the difference between open and closed questions. Results showed a significant increase from pre-intervention to post-visit patient activation scores (97). The intervention was valued by patients and added to their satisfaction with their care. Factors influencing their question-asking behaviour included their attitudes, social factors, and self-efficacy in question formulation (98).

One Canadian study evaluated the efficacy of two web-based education programs on communication among patients with chronic disease. Interventions were based on the PACE system: Prepare, Ask questions, Check understanding, Express concerns. The web-based program presented content as audio, stand-alone text, narrated text and presentations. Following the intervention, patients were more activated even in well-established doctor-patient relationships. (99).

### 9.4 Self-efficacy

Self-efficacy can be defined as “the confidence a person feels about performing a particular activity, including confidence in overcoming the barriers to performing that behaviour” (87). One study evaluated a communication program designed to improve transplant candidates’ communication self-efficacy. Development of the program was guided by a community advisory board who also provided personal accounts of their experience with transplantation for a video showing effective communication techniques. The final program included strategies for overcoming communicative barriers. Teaching strategies embedded in the modules were guided by social cognitive theory (‘observation of others’) as well as principles of adult learning. The study found significant pre–post increases in transplant knowledge and communication self-efficacy for participants. Decreases in perceived conversational difficulties were also observed (100).

## 10. Use of theories or frameworks

One study used the COM-B model to design an online communication program for gestational diabetes in Latina women (101). As described by the authors, the COM-B model and the Behaviour Change Wheel (BCW), deliver a theoretically based approach for intervention development. The COM-B model organizes domains into Capability, Opportunity, and Motivation-related factors: Capability includes both psychological and physical capability; Opportunity refers to factors in the environment or social setting that influence behaviour; and Motivation refers to beliefs and emotions/impulses that are not always consciously recognized, but often direct behaviour. The BCW was developed from a synthesis of 19 frameworks of behaviour change and includes three interrelated layers, the first uses the COM-B model to help identify the sources of behaviour that are selected for the intervention targets. The second layer provides direction to help identify intervention options, by a process of selecting from among nine intervention functions that could be applied to address behavioural barriers and leverage potential enablers. The outer layer of the BCW identifies seven policy options that can be used to help deliver the selected intervention functions. These include: service provision, communication/ marketing, fiscal measures, regulation, guidelines, legislation, and environmental/social planning (101).

A conceptual framework based on the concept of ‘concordance’, or shared understanding, between clinician and patient was used in one study. Rather than a one-way, top-down transmission, the concordance framework can be viewed as a two-way transactional communication process, reflecting patient responsibility for observation, self-reflection, and communication (49). The authors delivered a communication workshop to improve doctor-patient communication skills among a minority cancer patient population, focusing on concordance in several areas including: the patient's preference for information and involvement; the information conveyed by the physician; and the next steps the patients should take. This study also used the PACE framework to develop the workshop content. This framework includes four patient-level strategies for effective physician-patient communication: 1) Presenting detailed information about how they feel emotionally, any symptoms, history, reasons for visit etc.; 2) Asking questions including having a pre-set list of questions; 3) Checking their understanding of information that is given including asking the clinician to repeat or clarify information, and summarizing information back to the clinician to check understanding; and 4) Expressing any concerns (e.g., cultural beliefs) that may hinder treatment. (49, 58, 102, 103). Studies have identified that use of PACE is associated with improved self-management of chronic conditions (58), improved patient satisfaction with communication and (104), and greater adherence to treatment (102).

# RECOMMENDATIONS FOR THE CHECK-BACK ONLINE RESOURCE

Overall, findings from this review indicate that an online learning resource may support consumers to recognise the importance and value of checking their understanding, and provide them with the necessary skills and confidence to initiate teach-back during interactions with health professionals. To inform development of the consumer check-back module, key recommendations are:

- Interventions to improve communication skills should focus on building consumers’ awareness about the role they can play.
- Ensure computer aided learning is accessible to a wide range of consumers.
  - Font should be large and clear, with consistent layouts through.
  - Information should be brief and jargon-free.
- A mix of modalities should be used to deliver information, including illustrations, videos, text, animation and narration.
  - Videos can be used to demonstrate model behaviours, and provide advice and education.
  - Infographics and images should be “information-rich’” and use colour to denote meaning. Avoid stereotypical pictures or cartoons.
  - Use stories for delivering messages but combine with other strategies.
  - Include an interactive component.
- Add printed resources to support the messages in the online resource.
  - Include examples of question prompt lists (QPLs) but also provide advice about how to maximise clinician endorsement of their use.
  - Consider use of a Frequently Asked Question (FAQ) sheets that is visually appealing.
- Wherever possible, consider the needs of under-represented population groups.
  - For culturally diverse groups, use visual aids and show people from various cultural groups.
  - Include simple tutorials or clear instructions.
  - Include images plus transcripts of any video commentary to support people with a hearing impairment.
- Use approaches to empower and activate consumers and build self-efficacy in communication:
  - Use adult learning principles
  - Demonstrate target behaviours through video clips of peers
  - Develop question-asking skills

# REFERENCES

1. Grady A, Carey M, Bryant J, Sanson-Fisher R, Hobden B. A systematic review of patient-practitioner communication interventions involving treatment decisions. Patient Education & Counseling. 2017;100(2):199-211.

2. Mistiaen P, van Osch M, van Vliet L, Howick J, Bishop FL, Di Blasi Z, et al. The effect of patient-practitioner communication on pain: a systematic review. European Journal of Pain. 2016;20(5):675-88.

3. Farin E, Ullrich A, Nagl M. Health education literacy in patients with chronic musculoskeletal diseases: development of a new questionnaire and sociodemographic predictors. Health Education Research. 2013;28(6):1080-91.

4. Aldoory L. The Status of Health Literacy Research in Health Communication and Opportunities for Future Scholarship. Health Communication. 2017;32(2):211-8.

5. Okunrintemi V, Spatz ES, Di Capua P, Salami JA, Valero-Elizondo J, Warraich H, et al. Patient–Provider Communication and Health Outcomes Among Individuals With Atherosclerotic Cardiovascular Disease in the United States. Medical Expenditure Panel Survey 2010 to 2013. 2017;10(4).

6. Haskard Zolnierek KB, DiMatteo MR. Physician Communication and Patient Adherence to Treatment: A Meta-analysis. Medical care. 2009;47(8):826-34.

7. Harrison JD, Seymann G, Imershein S, Amin A, Afsarmanesh N, Uppington J, et al. The Impact of Unmet Communication and Education Needs on Neurosurgical Patient and Caregiver Experiences of Care: A Qualitative Exploratory Analysis. World neurosurgery. 2019;122:e1528-e35.

8. Newnham H, Barker A, Ritchie E, Hitchcock K, Gibbs H, Holton S. Discharge communication practices and healthcare provider and patient preferences, satisfaction and comprehension: A systematic review. International Journal for Quality in Health Care. 2017;29(6):752-68.

9. Street RL, Jr., Liu L, Farber NJ, Chen Y, Calvitti A, Zuest D, et al. Provider interaction with the electronic health record: the effects on patient-centered communication in medical encounters. Patient Education & Counseling. 2014;96(3):315-9.

10. Street RL. How clinician–patient communication contributes to health improvement: Modeling pathways from talk to outcome. Patient Education and Counseling. 2013;92(3):286-91.

11. Berger Z, Dembitzer A, Beach MC. Reason for hospital admission: a pilot study comparing patient statements with chart reports. Narrat Inq Bioeth. 2013;3(1):67-79.

12. Berger ZD, Boss EF, Beach MC. Communication behaviors and patient autonomy in hospital care: A qualitative study. Patient Educ Couns. 2017;100(8):1473-81.

13. Olson DP, Windish DM. Communication Discrepancies Between Physicians and Hospitalized Patients. Archives of Internal Medicine. 2010;170(15):1302-7.

14. Lipson-Smith R, Hyatt A, Murray A, Butow P, Hack TF, Jefford M, et al. Measuring recall of medical information in non-English-speaking people with cancer: A methodology. Health expectations : an international journal of public participation in health care and health policy. 2018;21(1):288-99.

15. Richard C, Glaser E, Lussier M-T. Communication and patient participation influencing patient recall of treatment discussions. Health Expectations. 2017;20(4):760-70.

16. Talevski J, A WS, Rasmussen B, Kemp G, A. B. Teach-back: A systematic review of implementation and impacts. PlosOne. 2020.

17. Ha Dinh TT, Bonner A, Clark R, Ramsbotham J, Hines S. The effectiveness of the teach-back method on adherence and self-management in health education for people with chronic disease: a systematic review. JBI Database of Systematic Reviews and Implementation Reports. 2016;14(1):210-47.

18. Dantic DE. A critical review of the effectiveness of ‘teach-back’ technique in teaching COPD patients self-management using respiratory inhalers. Health Education Journal. 2014;73(1):41-50.

19. Tran S, Bennett G, Richmond J, Nguyen T, Ryan M, Hong T, et al. 'Teach-back' is a simple communication tool that improves disease knowledge in people with chronic hepatitis B - a pilot randomized controlled study. BMC Public Health. 2019;19(1):1355.

20. Ha Dinh TT, Bonner A, Clark R, Ramsbotham J, Hines S. The effectiveness of the teach‐back method on adherence and self‐management in health education for people with chronic disease: a systematic review. JBI Database of Systematic Reviews and Implementation Reports. 2016;14(1):210-47.

21. Dantic DE. A critical review of the effectiveness of ‘teach-back’ technique in teaching COPD patients self-management using respiratory inhalers. Health Education Journal. 2013.

22. Talevski J, Wong Shee A, Rasmussen B, Kemp G, Beauchamp A. Teach-back: A systematic review of implementation and impacts. PLOS ONE. 2020;15(4):e0231350.

23. Australian Commission on Safety and Quality in Health Care. Health literacy: Taking action to improve safety and quality. Sydney, ACSQHC, . 2014.

24. Agency for Healthcare Research and Quality. Health Literacy Universal Precautions Toolkit 2nd Edition. Use the Teach-Back Method;Tool #5. 2017;<https://www.ahrq.gov/professionals/quality-patient-safety/quality-resources/tools/literacy-toolkit/healthlittoolkit2-tool5.html>.

25. Badaczewski A, Bauman LJ, Blank AE, Dreyer B, Abrams MA, Stein REK, et al. Relationship between Teach-back and patient-centered communication in primary care pediatric encounters. Patient Educ Couns. 2017;100(7):1345-52.

26. Samuels-Kalow M, Hardy E, Rhodes K, Mollen C. "Like a dialogue": Teach-back in the emergency department. Patient Educ Couns. 2016;99(4):549-54.

27. Ubel PA, Scherr KA, Fagerlin A. Empowerment Failure: How Shortcomings in Physician Communication Unwittingly Undermine Patient Autonomy. American Journal of Bioethics. 2017;17(11):31-9.

28. Feinberg I, Ogrodnick MM, Hendrick RC, Bates K, Johnson K, Wang B. Perception Versus Reality: The Use of Teach Back by Medical Residents. Health Lit Res Pract. 2019;3(2):e117-e26.

29. Noordman J, van Vliet L, Kaunang M, van den Muijsenbergh M, Boland G, van Dulmen S. Towards appropriate information provision for and decision-making with patients with limited health literacy in hospital-based palliative care in Western countries: a scoping review into available communication strategies and tools for healthcare providers. BMC Palliative Care. 2019;18(1):37.

30. Rozier RG, Horowitz AM, Podschun G. Dentist-patient communication techniques used in the United States: the results of a national survey. Journal of the American Dental Association (1939). 2011;142(5):518-30.

31. Berger ZD, Boss EF, Beach MC. Communication behaviors and patient autonomy in hospital care: A qualitative study. Patient Education and Counseling. 2017;100(8):1473-81.

32. Ubel PA, Scherr KA, Fagerlin A. Empowerment Failure: How Shortcomings in Physician Communication Unwittingly Undermine Patient Autonomy. Am J Bioeth. 2017;17(11):31-9.

33. Jager AJ, Wynia MK. Who gets a teach-back? patient-reported incidence of experiencing a teach-back. J Health Commun. 2012;17.

34. Stein PS, Aalboe JA, Savage MW, Scott AM. Strategies for communicating with older dental patients. Journal of the American Dental Association. 2014;145(2):159-64.

35. Paiva D, Abreu L, Azevedo A, Silva S. Patient-centered communication in type 2 diabetes: The facilitating and constraining factors in clinical encounters. Health Services Research. 2019;54(3):623-35.

36. Fink AS, Prochazka AV, Henderson WG, Bartenfeld D, Nyirenda C, Webb A, et al. Enhancement of surgical informed consent by addition of repeat back: a multicenter, randomized controlled clinical trial. Annals of surgery. 2010;252(1):27-36.

37. Kelly T, Arnold B, Surjan Y, Rinks M, Warren-Forward H. Radiation therapist health literacy training: A qualitative study exploring perceived barriers and attitudes. Radiography (London, England : 1995). 2020;26(4):294-301.

38. Morony S, Weir K, Duncan G, Biggs J, Nutbeam D, McCaffery K. Experiences of Teach-Back in a Telephone Health Service. Health literacy research and practice. 2017;1(4):e173-e81.

39. Klingbeil C, Gibson C. The Teach Back Project: A System-wide Evidence Based Practice Implementation. Journal of pediatric nursing. 2018;42:81-5.

40. D’Agostino TA, Atkinson TM, Latella LE, Rogers M, Morrissey D, DeRosa AP, et al. Promoting patient participation in healthcare interactions through communication skills training: A systematic review. Patient Education and Counseling. 2017;100(7):1247-57.

41. Tricco AC, Antony J, Zarin W, Strifler L, Ghassemi M, Ivory J, et al. A scoping review of rapid review methods. BMC Medicine. 2015;13(1):224.

42. Ahmed R, Bates BR. Patients’ fear of physicians and perceptions of physicians’ cultural competence in healthcare. Journal of Communication in Healthcare. 2017;10(1):55-60.

43. Joseph-Williams N, Elwyn G, Edwards A. Knowledge is not power for patients: A systematic review and thematic synthesis of patient-reported barriers and facilitators to shared decision making. Patient Education and Counseling. 2014;94(3):291-309.

44. Alders I, Smits C, Brand P, van Dulmen S. Does patient coaching make a difference in patient-physician communication during specialist consultations? A systematic review. Patient Education and Counseling. 2017;100(5):882-96.

45. Ondenge K, Renju J, Bonnington O, Moshabela M, Wamoyi J, Nyamukapa C, et al. 'I am treated well if I adhere to my HIV medication': putting patient-provider interactions in context through insights from qualitative research in five sub-Saharan African countries. Sexually Transmitted Infections. 2017;93(Suppl 3):07.

46. Talen MR, Muller-Held CF, Eshleman KG, Stephens L. Patients' communication with doctors: a randomized control study of a brief patient communication intervention. Families, Systems, & Health. 2011;29(3):171-83.

47. van Bruinessen IR, van Weel-Baumgarten EM, Gouw H, Zijlstra JM, Albada A, van Dulmen S. Barriers and facilitators to effective communication experienced by patients with malignant lymphoma at all stages after diagnosis. Psycho-Oncology. 2013;22(12):2807-14.

48. Brashers DE, Basinger ED, Rintamaki LS, Caughlin JP, Para M. Taking Control: The Efficacy and Durability of a Peer-Led Uncertainty Management Intervention for People Recently Diagnosed With HIV. Health Communication. 2017;32(1):11-21.

49. Bylund CL, Goytia EJ, D'Agostino TA, Bulone L, Horner J, Li Y, et al. Evaluation of a pilot communication skills training intervention for minority cancer patients. J Psychosoc Oncol. 2011;29(4):347-58.

50. Cooper LA, Roter DL, Carson KA, Bone LR, Larson SM, Miller ER, 3rd, et al. A randomized trial to improve patient-centered care and hypertension control in underserved primary care patients. Journal of General Internal Medicine. 2011;26(11):1297-304.

51. Epstein RM, Duberstein PR, Fenton JJ, Fiscella K, Hoerger M, Tancredi DJ, et al. Effect of a Patient-Centered Communication Intervention on Oncologist-Patient Communication, Quality of Life, and Health Care Utilization in Advanced Cancer: The VOICE Randomized Clinical Trial. JAMA Oncology. 2017;3(1):92-100.

52. Ab Malik N, Zhang J, Lam OL, Jin L, McGrath C. Effectiveness of computer-aided learning in oral health among patients and caregivers: a systematic review. Journal of the American Medical Informatics Association. 2017;24(1):209-17.

53. Salonen A, Ryhanen AM, Leino-Kilpi H. Educational benefits of Internet and computer-based programmes for prostate cancer patients: a systematic review. Patient Education & Counseling. 2014;94(1):10-9.

54. Feldman SR, Vrijens B, Gieler U, Piaserico S, Puig L, van de Kerkhof P. Treatment Adherence Intervention Studies in Dermatology and Guidance on How to Support Adherence. American Journal of Clinical Dermatology. 2017;18(2):253-71.

55. Jacobs RJ, Lou JQ, Ownby RL, Caballero J. A systematic review of eHealth interventions to improve health literacy. Health Informatics Journal. 2016;22(2):81-98.

56. Dol J, Delahunty-Pike A, Anwar Siani S, Campbell-Yeo M. eHealth interventions for parents in neonatal intensive care units: a systematic review. JBI Database Of Systematic Reviews And Implementation Reports. 2017;15(12):2981-3005.

57. Brijnath B, Protheroe J, Mahtani KR, Antoniades J. Do Web-based Mental Health Literacy Interventions Improve the Mental Health Literacy of Adult Consumers? Results From a Systematic Review. Journal of Medical Internet Research. 2016;18(6):e165.

58. Glaser E, Richard C, Lussier MT. The impact of a patient web communication intervention on reaching treatment suggested guidelines for chronic diseases: A randomized controlled trial. Patient Education & Counseling. 2017;100(11):2062-70.

59. Brooks C, Ballinger C, Nutbeam D, Adams J. The importance of building trust and tailoring interactions when meeting older adults' health literacy needs. Disability & Rehabilitation. 2017;39(23):2428-35.

60. Fredericks S, Martorella G, Catallo C. A systematic review of web-based educational interventions. Clinical Nursing Research. 2015;24(1):91-113.

61. Glaser J, Nouri S, Fernandez A, Sudore RL, Schillinger D, Klein-Fedyshin M, et al. Interventions to Improve Patient Comprehension in Informed Consent for Medical and Surgical Procedures: An Updated Systematic Review. Medical decision making : an international journal of the Society for Medical Decision Making. 2020;40(2):119-43.

62. Steinwachs DM, Roter DL, Skinner EA, Lehman AF, Fahey M, Cullen B, et al. A web-based program to empower patients who have schizophrenia to discuss quality of care with mental health providers. Psychiatr Serv. 2011;62(11):1296-302.

63. Brown-Johnson CG, Berrean B, Cataldo JK. Development and usability evaluation of the mHealth Tool for Lung Cancer (mHealth TLC): a virtual world health game for lung cancer patients. Patient Education & Counseling. 2015;98(4):506-11.

64. Pinheiro AP, Pocock RH, Dixon MD, Shaib WL, Ramalingam SS, Pentz RD. Using Metaphors to Explain Molecular Testing to Cancer Patients. Oncologist. 2017;22(4):445-9.

65. Krieger JL, Neil JM, Strekalova YA, Sarge MA. Linguistic Strategies for Improving Informed Consent in Clinical Trials Among Low Health Literacy Patients. Journal of the National Cancer Institute. 2017;109(3):03.

66. Licqurish SM, Cook OY, Pattuwage LP, Saunders C, Jefford M, Koczwara B, et al. Tools to facilitate communication during physician-patient consultations in cancer care: An overview of systematic reviews. CA: A Cancer Journal for Clinicians. 2019;69(6):497-520.

67. Barnes S, Gardiner C, Gott M, Payne S, Chady B, Small N, et al. Enhancing patient-professional communication about end-of-life issues in life-limiting conditions: a critical review of the literature. Journal of Pain & Symptom Management. 2012;44(6):866-79.

68. Belkora J, Miller M, Crawford B, Coyne K, Stauffer M, Buzaglo J, et al. Evaluation of question-listing at the Cancer Support Community. Translational behavioral medicine. 2013;3(2):162-71.

69. Hyatt A, Lipson-Smith R, Gough K, Butow P, Jefford M, Hack TF, et al. Culturally and linguistically diverse oncology patients' perspectives of consultation audio-recordings and question prompt lists. Psycho-Oncology. 2018;27(9):2180-8.

70. Moloczij N, Krishnasamy M, Butow P, Hack TF, Stafford L, Jefford M, et al. Barriers and facilitators to the implementation of audio-recordings and question prompt lists in cancer care consultations: A qualitative study. Patient Education & Counseling. 2017;100(6):1083-91.

71. Hjelmfors L, Stromberg A, Friedrichsen M, Sandgren A, Martensson J, Jaarsma T. Using co-design to develop an intervention to improve communication about the heart failure trajectory and end-of-life care. BMC Palliative Care. 2018;17(1):85.

72. Muscat DM, Chang EH-f, Thompson R, Cvejic E, Tracy M, Zadro J, et al. Evaluation of the Choosing Wisely Australia 5 Questions resource and a shared decision-making preparation video: protocol for an online experiment. BMJ Open. 2019;9(11):e033126.

73. Lockhart S, Dempsey AF, Pyrzanowski J, O'Leary ST, Barnard JG. Provider and Parent Perspectives on Enhanced Communication Tools for Human Papillomavirus Vaccine-Hesitant Parents. Academic pediatrics. 2018;18(7):776-82.

74. Finocchario-Kessler S, Catley D, Thomson D, Bradley-Ewing A, Berkley-Patton J, Goggin K. Patient communication tools to enhance ART adherence counseling in low and high resource settings. Patient Education & Counseling. 2012;89(1):163-70.

75. Arcia A, Suero-Tejeda N, Bales ME, Merrill JA, Yoon S, Woollen J, et al. Sometimes more is more: iterative participatory design of infographics for engagement of community members with varying levels of health literacy. Journal of the American Medical Informatics Association. 2016;23(1):174-83.

76. Poder TG, Carrier N, Bedard SK. Health Technology Assessment Unit Processes for the Validation of an Information Tool to Involve Patients in the Safety of Their Care. International Journal of Technology Assessment in Health Care. 2018;34(4):378-87.

77. Furuno Y, Sasajima H. Medical Comics as Tools to Aid in Obtaining Informed Consent for Stroke Care. Medicine. 2015;94(26):e1077.

78. McPherson AC, Knibbe TJ, Oake M, Swift JA, Browne N, Ball GDC, et al. "Fat is really a four-letter word": Exploring weight-related communication best practices in children with and without disabilities and their caregivers. Child: Care, Health & Development. 2018;44(4):636-43.

79. Nayak JG, Hartzler AL, Macleod LC, Izard JP, Dalkin BM, Gore JL. Relevance of graph literacy in the development of patient-centered communication tools. Patient Education & Counseling. 2016;99(3):448-54.

80. Trevena LJ, Zikmund-Fisher BJ, Edwards A, Gaissmaier W, Galesic M, Han PK, et al. Presenting quantitative information about decision outcomes: a risk communication primer for patient decision aid developers. BMC Medical Informatics & Decision Making. 2013;13 Suppl 2:S7.

81. Hartling L, Scott SD, Johnson DW, Bishop T, Klassen TP. A randomized controlled trial of storytelling as a communication tool. PLoS ONE [Electronic Resource]. 2013;8(10):e77800.

82. Jensen JD, King AJ, Carcioppolo N, Krakow M, Samadder NJ, Morgan S. Comparing tailored and narrative worksite interventions at increasing colonoscopy adherence in adults 50-75: a randomized controlled trial. Social Science & Medicine. 2014;104:31-40.

83. Koops van 't Jagt R, Tan SL, Hoeks J, Spoorenberg S, Reijneveld SA, de Winter AF, et al. Using Photo Stories to Support Doctor-Patient Communication: Evaluating a Communicative Health Literacy Intervention for Older Adults. Int J Environ Res Public Health. 2019;16(19):3726.

84. Koops van 't Jagt R, Hoeks JCJ, Duizer E, Baron M, Molina GB, Unger JB, et al. Sweet Temptations: How Does Reading a Fotonovela About Diabetes Affect Dutch Adults with Different Levels of Literacy? Health Communication. 2018;33(3):284-90.

85. Perez M, Sefko JA, Ksiazek D, Golla B, Casey C, Margenthaler JA, et al. A novel intervention using interactive technology and personal narratives to reduce cancer disparities: African American breast cancer survivor stories. Journal of Cancer Survivorship. 2014;8(1):21-30.

86. Torres DX, Lu WY, Uratsu CS, Sterling SA, Grant RW. Knowing How to Ask Good Questions: Comparing Latinos and Non-Latino Whites Enrolled in a Cardiovascular Disease Prevention Study. The Permanente journal. 2019;23.

87. Rochon D, Ross MW, Looney C, Nepal VP, Price AJ, Giordano TP. Communication strategies to improve HIV treatment adherence. Health Communication. 2011;26(5):461-7.

88. Lopez L, Tan-McGrory A, Horner G, Betancourt JR. Eliminating disparities among Latinos with type 2 diabetes: Effective eHealth strategies. Journal of Diabetes & its Complications. 2016;30(3):554-60.

89. Victorson D, Banas J, Smith J, Languido L, Shen E, Gutierrez S, et al. eSalud: designing and implementing culturally competent ehealth research with latino patient populations. American Journal of Public Health. 2014;104(12):2259-65.

90. Ferguson M, Leighton P, Brandreth M, Wharrad H. Development of a multimedia educational programme for first-time hearing aid users: a participatory design. International Journal of Audiology. 2018;57(8):600-9.

91. Kim SH, Lee A. Health-Literacy-Sensitive Diabetes Self-Management Interventions: A Systematic Review and Meta-Analysis. Worldviews on Evidence-Based Nursing. 2016;13(4):324-33.

92. Reiners F, Sturm J, Bouw LJW, Wouters EJM. Sociodemographic Factors Influencing the Use of eHealth in People with Chronic Diseases. International Journal of Environmental Research & Public Health [Electronic Resource]. 2019;16(4):21.

93. Castro EM, Van Regenmortel T, Vanhaecht K, Sermeus W, Van Hecke A. Patient empowerment, patient participation and patient-centeredness in hospital care: A concept analysis based on a literature review. Patient Education & Counseling. 2016;99(12):1923-39.

94. Kuijpers W, Groen WG, Aaronson NK, van Harten WH. A systematic review of web-based interventions for patient empowerment and physical activity in chronic diseases: relevance for cancer survivors. Journal of Medical Internet Research. 2013;15(2):e37.

95. Tannenbaum C, Martin P, Tamblyn R, Benedetti A, Ahmed S. Reduction of inappropriate benzodiazepine prescriptions among older adults through direct patient education: the EMPOWER cluster randomized trial. JAMA Internal Medicine. 2014;174(6):890-8.

96. Greene J, Hibbard JH. Why does patient activation matter? An examination of the relationships between patient activation and health-related outcomes. J Gen Intern Med. 2012;27(5):520-6.

97. Deen D, Lu W-H, Rothstein D, Santana L, Gold MR. Asking questions: The effect of a brief intervention in community health centers on patient activation. Patient Education and Counseling. 2011;84(2):257-60.

98. Lu W-H, Deen D, Rothstein D, Santana L, Gold MR. Activating Community Health Center Patients in Developing Question-Formulation Skills: A Qualitative Study. Health Education & Behavior. 2011;38(6):637-45.

99. Lussier MT, Richard C, Glaser E, Roberge D. The impact of a primary care e-communication intervention on the participation of chronic disease patients who had not reached guideline suggested treatment goals. Patient Education & Counseling. 2016;99(4):530-41.

100. Traino HM, West SM, Nonterah CW, Russell J, Yuen E. Communicating About Choices in Transplantation (COACH). Progress in Transplantation. 2017;27(1):31-8.

101. Handley MA, Harleman E, Gonzalez-Mendez E, Stotland NE, Althavale P, Fisher L, et al. Applying the COM-B model to creation of an IT-enabled health coaching and resource linkage program for low-income Latina moms with recent gestational diabetes: the STAR MAMA program. Implementation Science. 2016;11(1):73.

102. Cegala DJ, McClure L, Marinelli TM, Post DM. The effects of communication skills training on patients’ participation during medical interviews. Patient Education and Counseling. 2000;41(2):209-22.

103. Diefenbach M, Turner G, Carpenter KM, Sheldon LK, Mustian KM, Gerend MA, et al. Cancer and patient-physician communication. Journal of health communication. 2009;14 Suppl 1(Suppl 1):57-65.

104. Meropol NJ, Egleston BL, Buzaglo JS, Balshem A, Benson AB, 3rd, Cegala DJ, et al. A Web-based communication aid for patients with cancer: the CONNECT Study. Cancer. 2013;119(7):1437-45.
